# Neural regulation of tumor immunity: emerging opportunities to enhance cancer immunotherapy

**DOI:** 10.1038/s12276-026-01806-z

**Published:** 2026-08-13

**Authors:** Xu Chen, Meiyan Zou, Nina Li, Weiyao Feng, Pei Lin, Yunfan Lin, Xinyuan Zhao, Li Cui

**Affiliations:** 1 Stomatological Hospital, School of Stomatology, Southern Medical University, Guangzhou, Guangdong China; 2 School of Dentistry, University of California, Los Angeles, Los Angeles, CA USA

**Keywords:** Neuroimmunology, Immunosurveillance

## Abstract

The nervous system is a key regulator of cancer immunity, influencing tumor development and treatment response through neuroimmune interactions. Both peripheral and central circuits transmit neural signals that directly influence immune cell recruitment, activation, and effector function within the tumor microenvironment. Evidences indicate that sympathetic, parasympathetic, sensory neurons, and glial cells actively reshape the immune landscape through neurotransmitters and neuromodulators. Central neural circuits, such as catecholaminergic and stress-responsive pathways, further integrate psychological states and autonomic outflow to systemically reprogram immunity. However, how these diverse neural signals converge with tumor and immune cell interactions remains poorly defined. This Review synthesizes current advances across four dimensions: the roles of peripheral neurons and central neural circuits in cancer immunity; glial cell contributions to immunosuppression and tumor progression; reciprocal influences of tumor cells on neural remodeling; and neuron-independent neural signaling through immune-expressed adrenergic, cholinergic, and peptidergic receptors. We highlight that perineural invasion, tumor innervation heterogeneity, and receptor subtype-specific signaling are key factors in immune evasion and resistance to immune checkpoint inhibitor. By integrating these perspectives, this Review establishes the nervous system as a critical yet underexplored dimension of cancer immunology and proposes that context-defined and receptor-specific modulation of selected neuroimmune pathways may enhance immunotherapy.

## Nervous system shapes cancer immunity and treatment response

Cancer remains a substantial global health challenge, with traditional treatments often limited by recurrence and resistance. Recent advances in immunotherapy have shifted focus toward restoring host antitumor immunity; however, clinical efficacy varies due to immune evasion and suppressive tumor microenvironments. This Review explores the emerging role of neural regulation in cancer, highlighting how tumor innervation and neurotransmitter signaling influence immune cell dynamics and therapeutic responses. Researchers detail how adrenergic, cholinergic, and peptidergic signaling pathways, along with glial cell contributions, create a neuroimmune landscape that supports cancer progression. Key findings reveal that tumor-derived exosomes and neural remodeling promote immune evasion, whereas central neural circuits modulate systemic immunity. These insights underscore the potential of targeting neuroimmune pathways to enhance immunotherapy efficacy.

This summary was initially drafted using artificial intelligence, then revised and fact-checked by the author.

## Introduction

Cancer remains a major global health burden, with nearly 20 million new cases and 9.7 million deaths estimated worldwide in 2022 (ref. ^1^). Surgery, radiotherapy, and cytotoxic chemotherapy remain indispensable for reducing tumor burden, whereas molecularly targeted agents and antibody-based therapies have improved biological precision. Nevertheless, recurrence, metastatic dissemination, treatment-induced tissue injury, pathway redundancy, and resistant tumor states continue to limit long-term benefit^2–4^. Immunotherapy has shifted this therapeutic problem from direct tumor elimination toward restoration of host antitumor immunity, with immune checkpoint inhibitors, adoptive cell therapies, and therapeutic vaccines reshaping treatment across multiple malignancies^5^. However, its clinical efficacy remains uneven: productive responses require coordinated antigen presentation, T cell infiltration, and sustained effector function, whereas many tumors evolve immune evasion, immune exclusion, suppressive microenvironmental remodeling, and acquired resistance^6–8^. These response patterns have broadened the focus of cancer immunology from immune cells themselves to the physiological signals that condition immune competence within tumors. Neural regulation is increasingly recognized as one such influence, as tumor innervation, neurotransmitter, and neuropeptide signaling, glial activation, and stress-related neuroendocrine pathways can shape immune-cell recruitment, effector function, and therapeutic response^9,10^. Unlike vascular or stromal regulation, neural influences act with circuit-level precision, linking local immune dynamics to broader organismal states such as stress, metabolism, and circadian rhythm^11^.

Neurons within the tumor microenvironment (TME) are capable of exerting both pro-tumorigenic and antitumor functions, depending on their subtype, receptor usage, and location. Adrenergic, cholinergic, and peptidergic signaling influence immune cell balance, T lymphocyte metabolism, and myeloid populations^12,13^. Receptors such as β-adrenergic, nicotinic acetylcholine, and neuropeptide receptors mediate neural influence on immune cells^14^. Glial cells, including Schwann cells (SCs), astrocytes, and microglia, support tumor innervation and release signals that create suppressive niches^15,16^. SCs promote tumor migration and immune resistance, whereas astrocytes and microglia in brain tumors foster chronic inflammation and immune suppression^17–21^. Together, glial and neuronal inputs create a neuroimmune landscape that aids cancer progression.

**Fig. 1:**
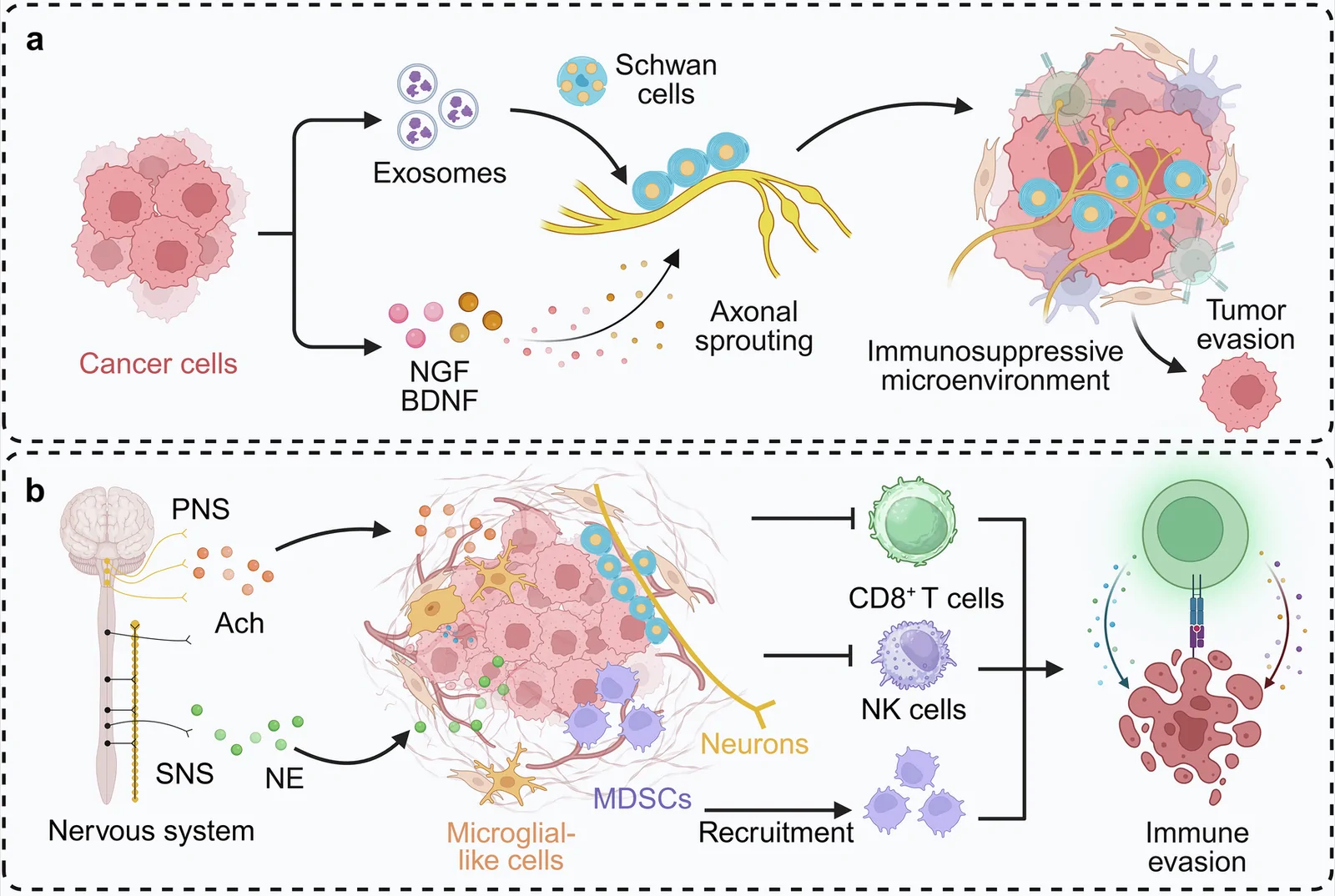
Tumor-driven neural remodeling and neuroimmune interactions promote immune evasion within the tumor microenvironment. **a** Cancer cells release extracellular vesicles, including exosomes, and neurotrophic factors such as nerve growth factor (NGF) and brain-derived neurotrophic factor (BDNF), which promote Schwann cell-associated axonal sprouting and nerve recruitment into tumor microenvironment. This tumor-driven neural remodeling increases intratumoral innervation and contributes to the formation of an immunosuppressive microenvironment that facilitates tumor evasion and dissemination. **b** Within the tumor microenvironment, autonomic neural inputs, including parasympathetic cholinergic signaling through acetylcholine (ACh) and sympathetic noradrenergic signaling through norepinephrine (NE), interact with cancer cells, neurons, Schwann cells, microglia-like macrophage-lineage cells, and immune cells. These neuroimmune interactions suppress CD8⁺ T cell and natural killer (NK) cell activity while promoting the recruitment and accumulation of myeloid-derived suppressor cells (MDSCs). Together, tumor-induced neural remodeling and neural regulation of immune cells converge to weaken antitumor immunity and promote immune evasion. PNS, parasympathetic nervous system; SNS, sympathetic nervous system.

These interactions are bidirectional, with tumor cells actively remodeling the surrounding neural architecture to promote their survival. By secreting neurotrophic factors such as nerve growth factor (NGF) and brain-derived neurotrophic factor (BDNF), cancer cells stimulate axonal sprouting, increase neural density, and activate glial cells, further aiding immune evasion^22–25^ (Fig. 1). Tumor-derived exosomes also recruit nerves by promoting axonogenesis. These nanosized extracellular vesicles (EVs) mediate intercellular communication and modulate immune responses through tumor-derived or neuron-derived bioactive molecules^26^. They stimulate neurite outgrowth and enhance sensory nerve sprouting, with exosomal EphrinB1 potentiating this effect. Inhibiting exosome release reduces tumor innervation, linking exosomes to nerve recruitment and cancer progression^27^. Exosomes from p53-deficient tumor cells promote neural recruitment more effectively than those from wild-type cells, increasing neurofilament abundance in dorsal root ganglia^28^. These findings highlight the role of tumor-derived exosomes in driving tumor innervation and the active involvement of the nervous system in the tumor–immune axis.

Neural activity integrates systemic signals into cancer immunity through central neural circuits, including stress-responsive pathways in the hypothalamus, brainstem catecholaminergic neurons, and limbic structures. These pathways modulate peripheral immunity by enhancing sympathetic drive, suppressing cytotoxic lymphocyte activity, and skewing macrophage and dendritic cell function^29–31^. This links emotional states, psychological stress, and circadian rhythms to tumor progression and immune surveillance, situating cancer immunity within broader neurophysiology^32–34^. Additionally, tumor-innervating nociceptor neurons form circuits linking peripheral tumors to brain regions, modulating neuronal activity and behavior, revealing a bidirectional tumor–brain neural axis^35^.

This Review synthesizes emerging evidence on the roles of neurons and glial cells in tumor immunity, with a particular focus on peripheral neural subsets — including sensory, sympathetic, and parasympathetic fibers — as well as central neural circuits. Conceptually, neuroimmune regulation in cancer can be organized into several interconnected levels. At the local level, peripheral nerves directly communicate with tumor, stromal, and immune cells within the TME. At the systemic level, central neural circuits regulate antitumor immunity indirectly through autonomic and neuroendocrine outputs, thereby linking stress, emotion, metabolism, and circadian rhythms to immune surveillance. In parallel, glial cells and tumor cells actively remodel neural architecture, generating reciprocal neuro–tumor circuits that reinforce immune suppression. Finally, tumor and immune cells can exploit neurotransmitter receptors or neural-like programs even in the absence of direct neuronal input. On the basis of this multilevel framework, this Review first discusses neuroimmune communication from homeostasis to disease progression and then examines how tumor-driven neural remodeling shapes immune suppression and cancer progression, followed by neuronal regulation of tumor immunity through peripheral and central circuits, glial cell contributions to immune evasion and TME plasticity, and neuron-independent neural signaling in tumor–immune evasion. We conclude by discussing key challenges and translational opportunities for targeting neuroimmune pathways to enhance immunotherapy efficacy.

## Neuroimmune communication from homeostasis to disease progression

The nervous and immune systems are intricately interconnected, forming a dynamic bidirectional network that maintains host defense, tissue integrity, and systemic homeostasis^36^. Neural inputs continuously monitor peripheral immune activity through both reflex circuits and soluble mediators, enabling rapid adjustment of immune responses to internal and external stimuli. Under physiological conditions, this communication safeguards immune balance by restraining excessive inflammation and promoting resolution^37^.

At steady state, parasympathetic and sympathetic pathways collaborate to regulate immune tone across organs. For example, a liver–brain–gut vagal reflex mediates peripheral tolerance by activating muscarinic acetylcholine receptors on intestinal antigen-presenting cells, thus promoting retinoic-acid production and regulatory T cell differentiation^38^. In the spleen, a vagus-dependent anti-inflammatory pathway activates acetylcholine-producing memory T cells, which suppress macrophage cytokine production via α7 nicotinic receptors, maintaining immune balance^39^.

Sympathetic neurons regulate immune function by releasing norepinephrine, which activates β-adrenergic receptors (β-ARs) on macrophages and lymphocytes, balancing protection and tolerance during stress or infection^40^. For instance, β2-AR signaling in muscularis macrophages promotes tissue protection, whereas β1-AR activation on CD8⁺ T cells induces exhaustion and suppresses antiviral immunity^41^. Additionally, neuroendocrine mediators such as glucocorticoids, catecholamines, and neuropeptides such as vasoactive intestinal peptide (VIP) and substance P (SP) influence immune responses by affecting cytokine production, antigen presentation, and lymphocyte trafficking^42^.

When neural–immune feedback is disrupted, protective mechanisms become maladaptive, leading to chronic inflammation and organ damage. For example, disruption of the cholinergic anti-inflammatory pathway contributes to conditions such as periodontitis and acute kidney injury by reducing α7 nAChR activity and promoting pro-inflammatory macrophage polarization^43,44^. Restoring vagal tone through interventions such as electroacupuncture or vagus nerve stimulation can reactivate this pathway and reduce inflammation. Similarly, imbalances in sympathetic output worsen metabolic disorders. In steatotic liver disease, VIP-producing neurons regulate fat absorption and IL-22 production by ILC3s, which protect the liver. Inhibiting VIP neurons or deleting Vipr2 in ILC3s restores IL-22 and alleviates hepatic steatosis^45^.

Building on the homeostatic neuroimmune circuits described earlier, tumorigenesis can progressively convert these protective feedback systems into maladaptive regulatory loops. Persistent tissue injury, chronic inflammation, metabolic stress, and tumor-derived mediators continuously stimulate neuronal and glial compartments, driving nerve sprouting, altered neurotransmitter and neuropeptide release, and the formation of perineural niches^46,47^. Under sustained signaling conditions, feedback mechanisms that normally constrain inflammation may become persistently activated, thereby reinforcing immunosuppressive pathways rather than restoring tissue equilibrium^48^. Consequently, neural signals that maintain immune balance in physiological settings can be rewired to suppress cytotoxic lymphocyte activity, promote immunosuppressive myeloid populations, and facilitate immune evasion. This transition from physiological regulation to pathological rewiring provides a mechanistic bridge linking baseline neuroimmune homeostasis to tumor-specific neuroimmune dysregulation.

## Tumor-driven neural remodeling shapes immune suppression and cancer progression

Tumor cells actively reprogram neuronal and glial circuits to construct a pro-tumorigenic niche, co-opting neural signaling to sustain proliferation, resist stress, and evade immune surveillance. This positions the nervous system as a dynamic component of the TME, in which neuronal activity and neurotransmitter dynamics promote cancer progression^46^.

**Fig. 2:**
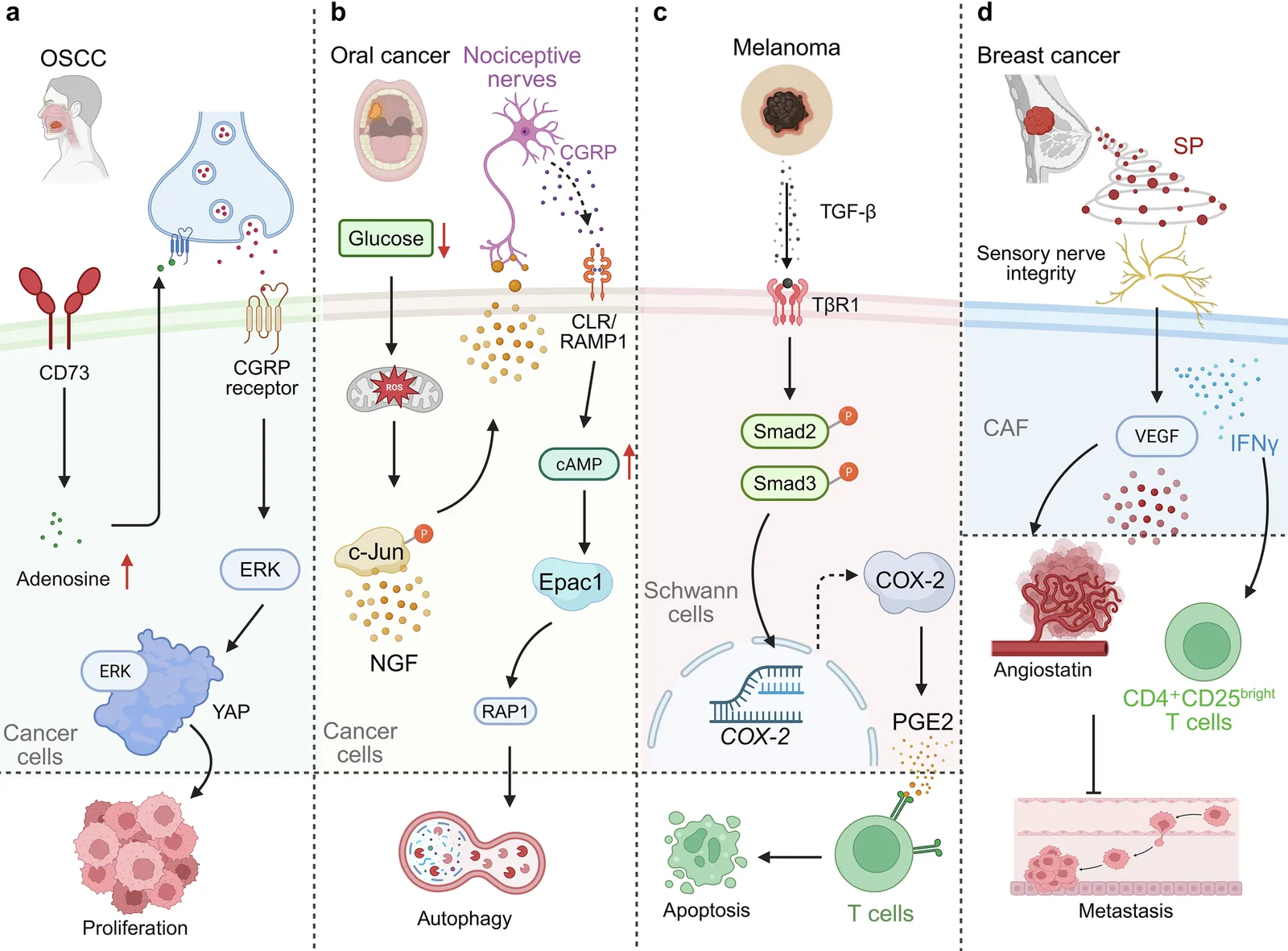
Tumor cells remodel neural circuits to regulate immune responses and promote immune evasion. **a** CD73 upregulation in oral squamous cell carcinoma (OSCC) drives adenosine accumulation, which overstimulates trigeminal A2AR to trigger calcitonin gene-related peptide (CGRP) release. CGRP activates ERK-YAP signaling in cancer cells, promoting progression. **b** In OSCC, tumor cells elevate metabolic stress and reactive oxygen species, activating Jun and upregulating nerve growth factor (NGF). NGF promotes nerve axonogenesis, whereas nociceptive neuron-derived CGRP further amplifies the Gαs–AC–cAMP–Epac1–RAP1 pathway in cancer cells, reinforcing autophagy and sustaining tumor growth. **c** In melanoma, tumor-derived transforming growth factor-beta (TGF-β) activates TβR1-mediated Smad2, Smad3, and Smad4 signaling and induces COX-2 expression in Schwann cells. Schwann cell-derived PGE2 suppresses CD8⁺ T cell activation via PD-1 signaling, weakening antitumor immunity. **d** In breast cancer with brain metastatic potential, substance P (SP) exerts an immunomodulatory and anti-metastatic effect by preserving sensory nerve integrity and reshaping cancer-associated fibroblast (CAF)-derived angiogenic signaling. SP promotes interferon-γ (IFNγ) production and CD4⁺CD25^bright^ T cell accumulation, while reducing myeloid-derived suppressor cell-mediated immunosuppression. VEGF, vascular endothelial growth factor.

In oral squamous cell carcinoma (OSCC), CD73-mediated adenosine accumulation activates A2AR on trigeminal neurons, stimulating calcitonin gene-related peptide (CGRP) release and ERK–YAP signaling to promote tumor growth. Blocking A2AR or CGRP suppresses tumor progression, showing how oncometabolites drive cancer growth through neural circuits^49^ (Fig. 2a). Under nutrient deprivation, OSCC cells induce ROS–Jun-dependent NGF secretion, triggering CGRP release and enhancing tumor cell survival by inhibiting mTOR–Raptor signaling. Metabolic therapies such as glycolytic or angiogenic inhibition can strengthen this loop, but CGRP antagonists, including FDA-approved antimigraine drugs, disrupt it and sensitize tumors to metabolic therapies^47^ (Fig. 2b).

Tumor and immune cells remodel neural circuits to enhance immunosuppression and reduce immunotherapy efficacy. Tumor-expressed ITGA5 binds fibronectin in SC matrices, reprogramming SCs to secrete NGF, promoting nerve growth, and impairing natural killer (NK) cell cytotoxicity. Blocking ITGA5 with cilengitide restores PD-1 immunotherapy response, highlighting ITGA5-driven neuroimmune remodeling as a therapeutic target^22^. Tumors also release leukemia inhibitory factor and galectin-3, activating hypothalamic nuclei to increase sympathetic outflow, suppress antitumor immunity, and accelerate progression^50^. In colon cancer, SCs enhance tumor progression by secreting IL-6 via Jun reprogramming, with Jun ablation suppressing this effect^51^. Tumor-derived transforming growth factor-beta (TGF-β) activates SCs to produce PGE, which inhibits T cell proliferation and induces exhaustion, effects reversed by COX-2 or TGF-β inhibition^52^ (Fig. 2c). Tumor-derived EVs recruit TRPV1⁺ sensory neurons to secrete IL-6 and neuropeptides, promoting myeloid-derived suppressor cell (MDSC) differentiation and CD8⁺ T cell exhaustion in head and neck squamous cell carcinoma (HNSCC)^53^. Tumor-induced DKK1 expression in peripheral nerves impairs T cell activity and promotes MDSC recruitment, with DKK1 neutralization restoring immune surveillance^54^.

Neural rewiring further contributes to immune escape and therapy resistance. In glioma, tumor-induced glutamatergic hyperexcitability and synaptogenesis, mediated by thrombospondin-1, impair CD8⁺ T cell function and promote anti-inflammatory macrophage accumulation. Disrupting glutamatergic signaling or deleting thrombospondin-1 enhances immune responses and improves immunotherapy efficacy^55^. In pancreatic ductal adenocarcinoma (PDAC), IL-6 induces PVT1 expression in SCs, promoting kynurenine-mediated immunosuppression, while depleting SC-specific PVT1 restores immune surveillance and sensitizes tumors to immune checkpoint inhibitor (ICI)^56^. Perineural invasion and cancer-induced nerve injury drive resistance to PD-1 blockade by triggering demyelination, which leads to chronic inflammation and T cell exhaustion. Denervation or blocking IL-6 and interferon signaling restores antitumor immunity, highlighting cancer-induced nerve injury as a key mechanism underlying checkpoint resistance^57^.

Although most tumor-derived mediators dampen antitumor immunity, exceptions exist. SP from supporting or infiltrating immune cells modulates the neuroimmune microenvironment by reducing MDSC infiltration and enhancing CD4⁺Cd25^bright^ T cell accumulation, promoting IFNγ secretion. In breast cancer with brain metastatic potential, SP preserves sensory nerve integrity and alters angiogenic signaling from cancer-associated fibroblasts (CAFs). Combined with radiotherapy, SP enhances antitumor immunity, induces complete tumor regression in some animals, and suppresses metastasis^58^ (Fig. 2d).

## Neuronal regulation of tumor immunity in peripheral and central circuits

Neural dysregulation is a key regulator of antitumor immunity. In pancreatic cancer, SCs and immunosuppressive macrophages colocalize around nerves, aiding tumor cell migration^59^. In colorectal cancer (CRC), perineural invasion correlates with reduced lymphocytic infiltration and poor prognosis, highlighting neural invasion as a critical factor in immune evasion^60^. Neurons in both the peripheral and central nervous systems have an active role in modulating tumor biology by releasing neurotransmitters and neuropeptides that reshape TME, influencing immune cell function^61^. Peripheral neurons, including sensory, sympathetic, and parasympathetic types, interact with tumor and immune cells to drive context-dependent outcomes^62^. For example, sciatic nerve stimulation enhances antitumor immunity in triple-negative breast cancer by activating NK cells, which synergize with PD-1 blockade^63^. Central neurons also influence tumor immunity through autonomic pathways, with stress-responsive brain circuits shaping peripheral immune dynamics^64^. These findings highlight the potential of targeting neuroimmune pathways for cancer therapy.

### Sensory neurons as modulators of tumor immunity and immunotherapy

Sensory neurons are increasingly recognized as active participants in shaping the tumor–immune landscape. Through intrinsic molecular programs as well as the release of neurotransmitters and neuropeptides, these neurons exert multifaceted control over immune cell infiltration, activation, and functional states within TME. Such regulation profoundly influences tumor progression and responsiveness to immunotherapy^65^.

#### Sensory neuron-intrinsic programs shape tumor immunity and therapeutic responses

**Fig. 3:**
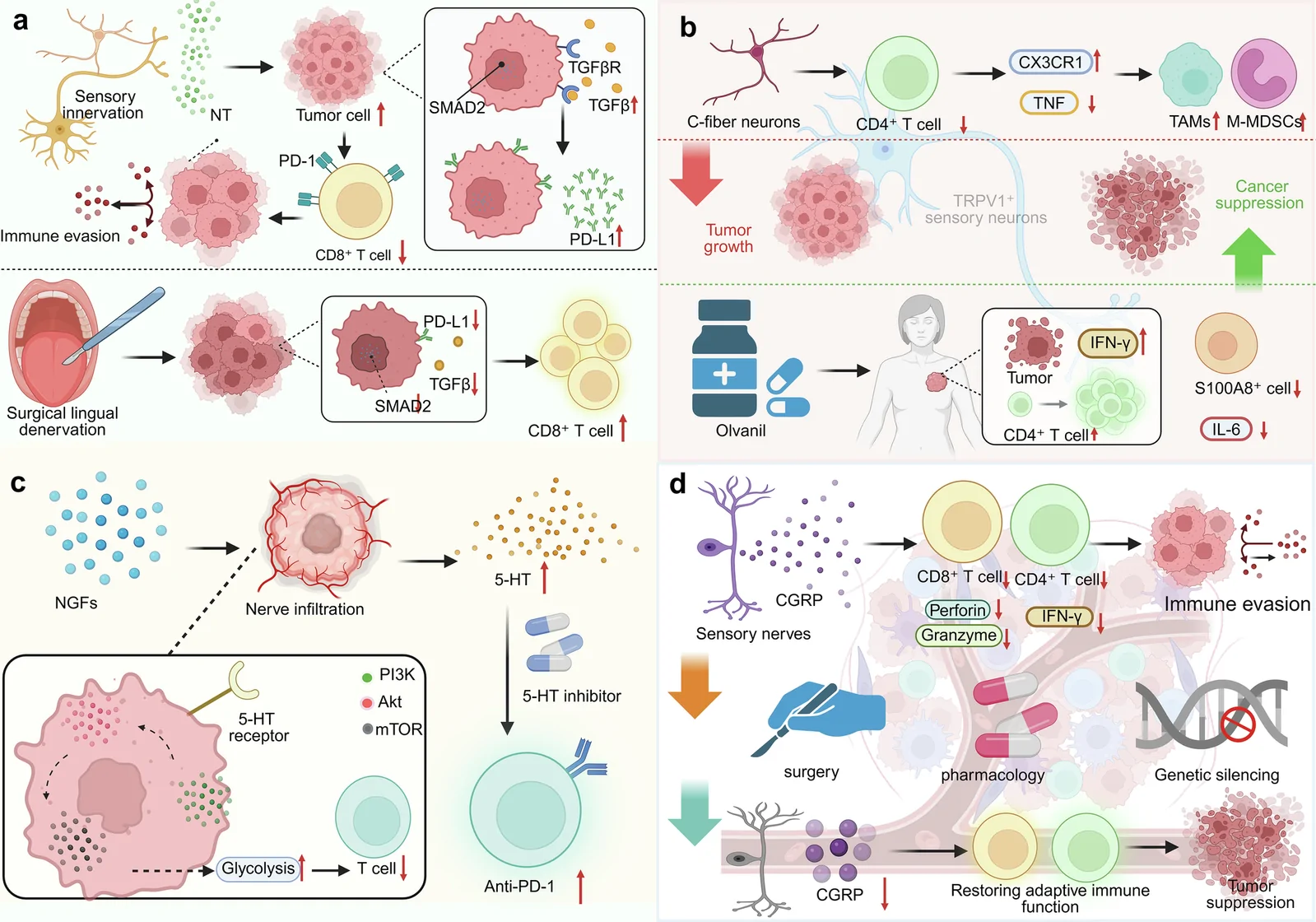
Sensory neuron–tumor crosstalk drives immune evasion and tumor progression, with therapeutic implications. **a** Sensory innervation of tumors releases neurotransmitters (NTs) that promote immune evasion by enhancing PD-L1 and transforming growth factor-beta (TGF-β) signaling in cancer cells, leading to suppression of CD8⁺ T cell activity via SMAD2-dependent pathways. Surgical lingual denervation reduces PD-L1 and TGF-β expression, restoring CD8⁺ T cell function. **b** C-fiber neurons regulate immune responses through TRPV1^+^ sensory circuits. Their activation increases CX3CR1 expression and decreases tumor necrosis factor-alpha (TNFα) levels in CD4^+^ T cells, thus limiting the activation of tumor-associated macrophages (TAMs) and M-myeloid-derived suppressor cells (MDSCs), which in turn promotes tumor growth. Pharmacological activation with olvanil enhances CD8^+^ T cell responses and interferon-γ (IFNγ) production systemically and intratumorally, while reducing the infiltration of pro-inflammatory IL-6 and S100A8^+^ cells, leading to tumor suppression. **c** Nerve growth factor (NGF) promotes nerve sprouting of axons into tumors, increasing 5-hydroxytryptamine (5-HT) release. Activation of 5-HT receptors in cancer cells triggers PI3K–Akt–mTOR signaling, driving glycolysis and T cell suppression. Inhibition of 5-HT signaling sensitizes tumors to anti-PD-1 therapy. **d** Sensory nerve-derived calcitonin gene-related peptide (CGRP) suppresses CD8⁺ T cell cytotoxicity (perforin and granzyme) and CD4⁺ T cell IFNγ production, enabling immune escape. Both surgical denervation and pharmacological or genetic silencing of CGRP restore adaptive immunity and suppress tumor growth.

Sensory neurons modulate tumor immunity through molecular programs and receptor-mediated signaling, reshaping the TME and regulating immune cell infiltration, effector activity, and myeloid cell polarization, which influence tumor progression and immunotherapy outcomes^66^. In melanoma, sensory innervation suppresses antitumor immunity by limiting T cell activation and tertiary lymphoid structure formation, whereas denervation enhances endothelial maturation, facilitates lymphocyte recruitment, and restores CD8⁺ T cell-dependent tumor control^67^. Similarly, in OSCC, sensory neurons activate TGF-β–SMAD2 signaling and upregulate PD-L1 expression in tumor cells, whereas surgical denervation mitigates these effects and enhances anti-PD-1 therapy efficacy^68^ (Fig. 3a).

Sensory neurons releasing CGRP shape the immune landscape in OSCC. Loss of CGRP signaling suppresses tumor growth and enhances intratumoral infiltration of CD4⁺ and CD8⁺ T cells as well as NK cells^69,70^. CGRP signaling drives the exhaustion of cytotoxic CD8⁺ T cells, promoting tumor growth, but genetic or pharmacological silencing of nociceptors or blockade of the CGRP receptor (RAMP1) restores T cell functionality and suppresses tumor progression, revealing a neural–immune axis that impedes effective cancer immunity^71^.

Persistent activation of TRPV1⁺ sensory neurons importantly impacts tumor progression by reshaping the immune landscape within the TME. Chemogenetic stimulation of C-fiber neurons suppresses CD4⁺ T cell infiltration and promotes an immunosuppressive myeloid phenotype, facilitating tumor-associated macrophage and M-MDSC accumulation^72^. By contrast, selective activation of TRPV1⁺ sensory nerves by olvanil inhibits breast cancer metastasis by enhancing CD8⁺ T cell activity and IFNγ production, while reducing IL-6 and S100A8⁺ myeloid infiltration^73^ (Fig. 3b). These findings highlight the bidirectional role of TRPV1 signaling in cancer, with its immunological outcome dependent on the intensity, duration, and spatial context of neural activation, emphasizing the need to fine-tune TRPV1 signaling for optimal antitumor efficacy.

#### Sensory neuron-derived neurotransmitters and neuropeptides as regulators of tumor immunity

Sensory neurons influence tumor immunity by releasing neurotransmitters and neuropeptides that act directly on tumor and immune cells, affecting cellular metabolism, immune function, and immunotherapy response^74^. For example, nerve-derived NGF promotes axonogenesis, whereas elevated 5-hydroxytryptamine (5-HT) enhances glycolysis in tumor cells via the PI3K-Akt-mTOR pathway, leading to metabolic reprogramming and increased immunosuppression in the TME. Neutralizing 5-HT signaling restores metabolic balance and improves PD-1 blockade efficacy^75^ (Fig. 3c). Although 5-HT in the TME can be produced by tumor or immune cells, its neuronal contribution is minimal^76^. Additionally, neurotransmitters can form functional neuro–tumor circuits that directly promote tumor progression. In gastric cancer, NGF-driven expansion of CGRP⁺ nociceptor neurons forms a CGRP–RAMP1 loop, enhancing calcium influx and accelerating growth and metastasis. Disrupting this pathway via sensory denervation or CGRP inhibition suppresses tumor progression and prolongs survival^77^.

Neuronal secreted factors reshape antitumor immunity by reprogramming immune subsets. In melanoma, nociceptor-derived CGRP induces CD8⁺ T cell exhaustion, and inhibiting CGRP–RAMP1 signaling restores cytotoxic function and improves survival. The proportion of RAMP1⁺ CD8⁺ T cells correlates with T cell exhaustion and poor clinical outcomes in both murine and human melanomas^71^. Similarly, in HNSCC, CGRP from sensory nerves reduces CD8⁺ T cell cytotoxicity and T_H_1 polarization, promoting immune escape^78^ (Fig. 3d). Tumor-derived EVs recruit sensory neurons to secrete IL-6 and neuropeptides, promoting MDSC differentiation and inducing CD8⁺ T cell exhaustion via TRPV1⁺ signaling. Disrupting this neuroimmune interaction restores adaptive immunity and limits tumor growth^53^.

Beyond modulating T cell responses, CGRP signaling regulates a broader immune network within the TME. In esophageal squamous cell carcinoma, sensory neuron-derived CGRP induces an immunosuppressive subset of RAMP1⁺ B cells, which secrete cytokines that suppress CD8⁺ T cell cytotoxicity, reducing antitumor immunity. Inhibition of CGRP–RAMP1 signaling reprograms B cell function and restores PD-1 blockade responsiveness^79^. Similarly, in PDAC, nociceptor neurons interact with CAFs through CGRP and NGF signaling to downregulate IL-15, reducing NK cell recruitment and cytotoxicity. Increased nociceptive innervation is associated with reduced NK cell infiltration, heightened pain, and poorer survival, identifying neural activity as an independent prognostic factor^80^ (Table 1).

**Table 1:** Sensory neuron-mediated neuroimmune mechanisms in tumor progression and immunotherapy.

Neuron type	Neurotransmitter/neuropeptide	Target cell	Immune modulation mechanism	Tumor type	Model system and evidence type	Therapeutic potential	Ref.
Sensory neurons	–	T cells, B cells	Inhibiting T cell activation, leukocyte recruitment, and tertiary lymphoid structure (TLS) assembly	Melanoma	Mouse melanoma model; in vivo sensory denervation; preclinical evidence	Sensory denervation enhances antitumor immunity	^45^
	–	OSCC cells, CD8⁺ T cells	Promoting OSCC cell TGFβ–SMAD2 axis and PD-L1 expression	OSCC	Mouse OSCC model; in vivo lingual denervation and anti-PD-1 treatment; preclinical evidence	Lingual denervation synergizes with anti-PD-1 to restore antitumor immunity	^46^
	CGRP	CD8⁺ T cells, CD4⁺ T cells, NK cells	Inhibiting antitumor–immune cell infiltration	HNSCC	Human HNSCC specimens + CGRP-deficient mouse model; translational evidence	Using nervous system drugs to treat cancer	^48^
TRPV1 sensory neurons (C-fibers)	–	CD4⁺ T cells, TAM, M-MDSCs	Suppressing CD4⁺ cell infiltration, skewing macrophages toward an immunosuppressive phenotype	–	Trpv1-Cre mouse tumor model; in vivo TRPV1⁺ neuron activation; mechanistic evidence	Limiting tumor progression	^22^
	–	CD8⁺ T cells	Enhancing CD8⁺ T cell responses, reducing IL-6 and S100A8 infiltration, reshaping TME	Breast cancer	Mouse breast cancer metastasis model; in vivo TRPV1 activation; therapeutic evidence	To inhibit breast cancer metastasis	^50^
Sensory neurons	5-HT	Non-small-cell lung cancer (NSCLC) cells	Enhancing glycolysis	NSCLC	Human NSCLC specimens + mouse tumor model; 5-HT and PD-1 intervention; translational evidence	To improve anti-PD-1 efficacy	^52^
Nociceptor neurons	CGRP	GC cells	Driving CGRP–RAMP1 circuit	Gastric cancer	Mouse gastric cancer model + human GC validation; chemogenetic,optogenetic and pharmacological evidence	Suppressing tumor growth and prolongs survival	^54^
Nociceptor neurons	CGRP	CD8⁺ T cells	Driving CD8⁺ T cell exhaustion and impairing cytotoxicity	Melanoma	Mouse melanoma model + human melanoma validation; translational evidence	Silencing nociceptors or blocking CGRP–RAMP1 to limit tumor growth	^49^
	CGRP	CD8⁺ T cells, T_H_1 cells	Impairing CD8⁺ T cell cytotoxicity and reducing T_H_1 cell responses	HNSCC	Human HNSCC samples + murine HNSCC model; in vitro and in vivo mechanistic evidence	–	^55^
	SP	CD8⁺ T cells	sEVs activate nociceptor neurons, enhancing MDSC infiltration	HNSCC Melanoma	DRG neuron–tumor sEV co-culture + mouse tumor model; in vitro and vivo mechanistic evidence	Disrupting detrimental neuroimmune crosstalk	^56^
	CGRP	B cells, CD8⁺ T cells	Inducing regulatory RAMP1⁺ B cells, suppressing CD8⁺ T cell cytotoxicity	ESCC	Human clinical cohort + preclinical model; translational evidence	Boosting anti-PD-1 efficacy	^57^
	CGRP	CAFs, NK cells	Downregulating IL-15, suppressing NK infiltration and cytotoxicity	PDAC	Human PDAC specimens + mouse PDAC model; CGRP and NGF mechanistic validation; translational evidence	–	^58^

*CAF* cancer-associated fibroblast, *CGRP* calcitonin gene-related peptide, *DRG* dorsal root ganglion, *ESCC* esophageal squamous cell carcinoma, *HNSCC* head and neck squamous cell carcinoma, *MDSC* myeloid-derived suppressor cell, *NK* natural killer, *OSCC* oral squamous cell carcinoma, *PDAC* pancreatic ductal adenocarcinoma, *sEV* Small extracellular vesicles, *SP* substance P, *TAM* tumor-associated macrophage, *TGF-ß* transforming growth factor-beta, *TME* tumor microenvironment, *5-HT 5*-hydroxytryptamine.

### SNS signaling as a multifaceted regulator of tumor biology and immunity

#### SNS regulation of tumor growth, metabolism, and metastasis

Sympathetic nervous system (SNS) activity is a key regulator of tumor biology, influencing growth, metabolism, and metastasis through adrenergic signaling. In small-cell lung cancer (SCLC), β2-AR activation promotes tumor cell proliferation via protein kinase A signaling, whereas β2-AR inhibition suppresses growth and prolongs survival^81^. SCLC cells also engage in direct neuron–cancer communication: vagal innervation facilitates primary tumor progression, and cortical glutamatergic and GABAergic neurons drive proliferation. Disrupting glutamate signaling abrogates this neural support, showing how SCLC hijacks neuronal circuits for sustained malignancy^82,83^.

**Fig. 4:**
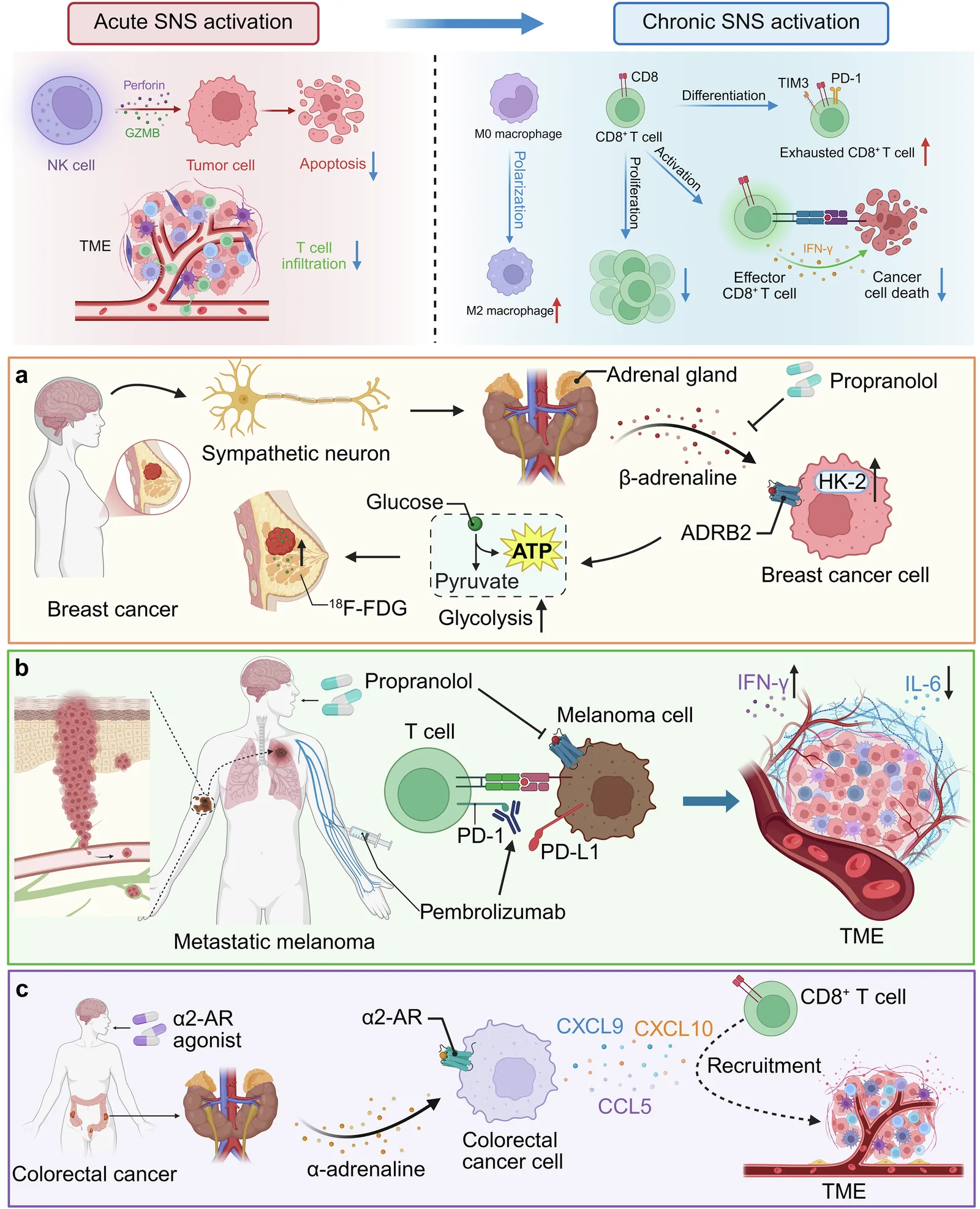
Adrenergic signaling modulates tumor metabolism and immune responses across cancer types. A temporal axis from acute to chronic sympathetic nervous system (SNS) activation is incorporated to highlight the dynamic transition from early impairment of natural killer (NK) cell cytotoxicity and T cell infiltration to later tumor microenvironment (TME) remodeling characterized by CD8^+^ T cell exhaustion and M2 macrophage polarization. **a** In breast cancer, sympathetic neuronal activation stimulates the adrenal gland to release β-adrenaline, which binds β2-adrenergic receptors (ARs) on tumor cells. This enhances hexokinase-2 (HK2) activity, glycolytic flux, and ^18^F-FDG uptake, thereby fueling tumor growth. Propranolol, a non-selective β-adrenergic blocker, suppresses this metabolic reprogramming. **b** In metastatic melanoma, propranolol enhances the efficacy of PD-1 blockade (pembrolizumab) by promoting T cell-mediated antitumor immunity. β-Adrenergic inhibition restores interferon-γ (IFNγ) production, reduces IL-6, and strengthens immune-mediated control of the TME. **c** In colorectal cancer, α2-AR signaling driven by α-adrenaline influences tumor–immune interactions. Pharmacological activation of α2-AR enhances secretion of chemokines (CXCL9, CXCL10, and CCL5) by tumor cells, facilitating CD8⁺ T cell recruitment into the TME and promoting antitumor responses. GZMB, granzyme B.

In breast cancer, β2-AR activation sustains glycolytic metabolism by stabilizing hexokinase-2 (HK2) expression, whereas propranolol reduces HK2 levels and ¹⁸F-FDG uptake^84^ (Fig. 4a). Chronic SNS activation promotes lymphatic metastasis by remodeling tumor-associated vasculature via VEGFC release and COX-2-dependent macrophage signaling. Blocking SNS signaling reduces metastatic spread^85^. In CRC, norepinephrine triggers CAFs to secrete NGF, enhancing sympathetic innervation and norepinephrine accumulation. This feedback accelerates CRC progression through YAP activation, and NGF further enhances growth via PI3K-AKT signaling. Targeting Trk signaling disrupts this synergy and limits tumor progression^86^.

#### SNS regulation of tumor immunity and immunotherapy

SNS signaling has a critical role in tumor immunity and therapeutic efficacy^87^. SNS activity reshapes the TME by reprogramming lymphoid and myeloid compartments, suppressing antitumor responses, and limiting ICI responsiveness^88^. In clinical studies, inhibiting β-adrenergic signaling has been shown to enhance antitumor immunity and survival. A retrospective study found that β-blocker use in advanced solid tumors, particularly urothelial carcinoma, correlated with improved disease control. Ongoing trials are exploring the immunomodulatory potential of β-blockers across cancer types. For example, in metastatic melanoma, propranolol combined with pembrolizumab achieved a 78% objective response rate, increasing IFNγ and decreasing IL-6, suggesting enhanced antitumor immunity (NCT03384836)^89^ (Fig. 4b). Other trials in CRC and breast cancer are investigating the ability of propranolol to reverse immune suppression and enhance ICI efficacy (NCT00888797 and NCT01847001). Despite these promising results, clinical findings are inconsistent, likely due to differences in tumor type, study design, and β-blocker characteristics. Future biomarker-guided trials are needed to define optimal strategies and clarify the mechanistic role of neuroimmune modulation.

Preclinical studies support these findings. In murine melanoma, β-AR blockade, particularly β2-AR inhibition, enhances PD-1 blockade efficacy and synergizes with high-dose IL-2 therapy^80^. In PDAC, SNS activity contributes to immune evasion through neuronal remodeling. Denervation triggers a pro-inflammatory TME and enhances ICI efficacy, combining sympathetic denervation and taxane treatment potentiating tumor regression^90^.

At the cellular level, CD8⁺ T cells are key mediators of adrenergic control, with SNS activation impairing antitumor immunity. Adrenergic stimulation restricts T cell trafficking via vasoconstriction-induced hypoxia, limiting infiltration independently of β-receptor engagement^91^. β2-AR signaling suppresses CD8⁺ T cell function by reducing T cell receptor-driven cytokine production and cytotoxicity, effects reversible by β2 antagonism^92^. Exhausted CD8⁺ T cells in multiple cancers show increased expression of Gαs-coupled G protein-coupled receptors, including β1AR and β2AR, enforcing dysfunction through the Gαs–PKA axis^93^. Chronic β-AR stimulation suppresses antigen-specific proliferation, IFNγ secretion, and cytotoxicity, impairing the efficacy of anti-PD-1 and anti-4-1BB therapies. β-Blockers restore T cell function and synergize with ICIs^94^. Sustained β1-AR and β2-AR activation accelerates exhaustion of tumor-infiltrating lymphocytes, whereas β-blocker restores progenitor-like and cytotoxic phenotypes, synergizing with ICIs^95^. At the metabolic level, β2-AR signaling disrupts glycolysis and mitochondrial reprogramming, increasing exhaustion markers. β-Blockers such as propranolol restore CD28 expression and reactivation of exhausted T cells^87,96^.

SNS signaling also influences broader immune cell plasticity. β1-AR activity promotes FOXP3⁺CXCR3⁺ mucosa-associated invariant T cells in hepatocellular carcinoma, enhancing their immunosuppressive function^97^. Chronic stress promotes breast cancer progression through β2-AR-dependent M2 macrophage polarization^91^. Acute stress suppresses NK cell cytotoxicity via β-adrenergic activation, facilitating metastasis, which is reversed by blocking SNS signaling^98^. In the myeloid lineage, SNS signaling through α-ARs and β2-ARs regulates MDSC expansion. β2-AR activation reprograms MDSCs toward immunosuppression, increasing PGE2, PD-L1, and arginase-I expression^99,100^. Inhibiting β-adrenergic signaling normalizes myeloid differentiation, reducing MDSC accumulation and restoring effector immune responses^101^.

Collectively, these clinical and experimental findings establish SNS signaling as a central regulator of tumor–immune interactions. Through β-adrenergic control of T cell activation, metabolism, and exhaustion, as well as myeloid and innate immune reprogramming, the SNS dictates the immune tone of the TME and the efficacy of immunotherapy. Targeting adrenergic signaling, therefore, represents a rational and promising strategy to reprogram the immunosuppressive TME, enhance immune responsiveness, and improve patient outcomes across cancer types.

#### Context-dependent antitumor roles of sympathetic signaling

Sympathetic signaling can exert both immunostimulatory and tumor-suppressive effects depending on receptor subtype and microenvironmental context. In PDAC, sympathetic innervation arises from both passive fiber incorporation and active axonal sprouting into tumor-adjacent tissues. Ablation of these nerves accelerates tumor growth by expanding CD163⁺ immunosuppressive macrophages within the TME^102^. Similarly, in CRC, loss of sympathetic innervation promotes tumor progression by impairing immune surveillance. Norepinephrine–α2-AR signaling in sympathetic nerves induces chemokine expression in tumor cells, recruiting CD8⁺ T cells. Neurodegeneration reduces cytotoxic T cell infiltration and facilitates malignancy^103^ (Fig. 4c). Pharmacological activation of α2-ARs elicits antitumor immunity across various immunocompetent and ICI-resistant models, enhancing T cell infiltration and reducing apoptotic myeloid suppressor cells. α2-AR stimulation directly activates macrophages, boosting antigen presentation and coordinating innate and adaptive immune responses^104^.

The dual nature of SNS signaling in cancer immunity reflects receptor subtype specificity, tumor context, and immune composition. β-Adrenergic stress signaling impairs T cell priming, drives exhaustion, and sustains immunosuppressive myeloid populations, limiting ICI efficacy. By contrast, α2-adrenergic inputs promote immune surveillance by enhancing T cell recruitment and macrophage activation, even in immunotherapy-resistant tumors^103^. These observations suggest that SNS activity acts as a context-dependent rheostat of immunity. Chronic β-adrenergic activation promotes immunosuppression, whereas α2-adrenergic signaling sustains immune vigilance. Therapeutically, this duality supports precision neuroimmunology strategies that selectively block β-driven suppression while amplifying α2-mediated stimulation, restoring neuroimmune balance for durable antitumor immunity.

### Parasympathetic regulation of tumor immunity through vagal and cholinergic signaling

**Fig. 5:**
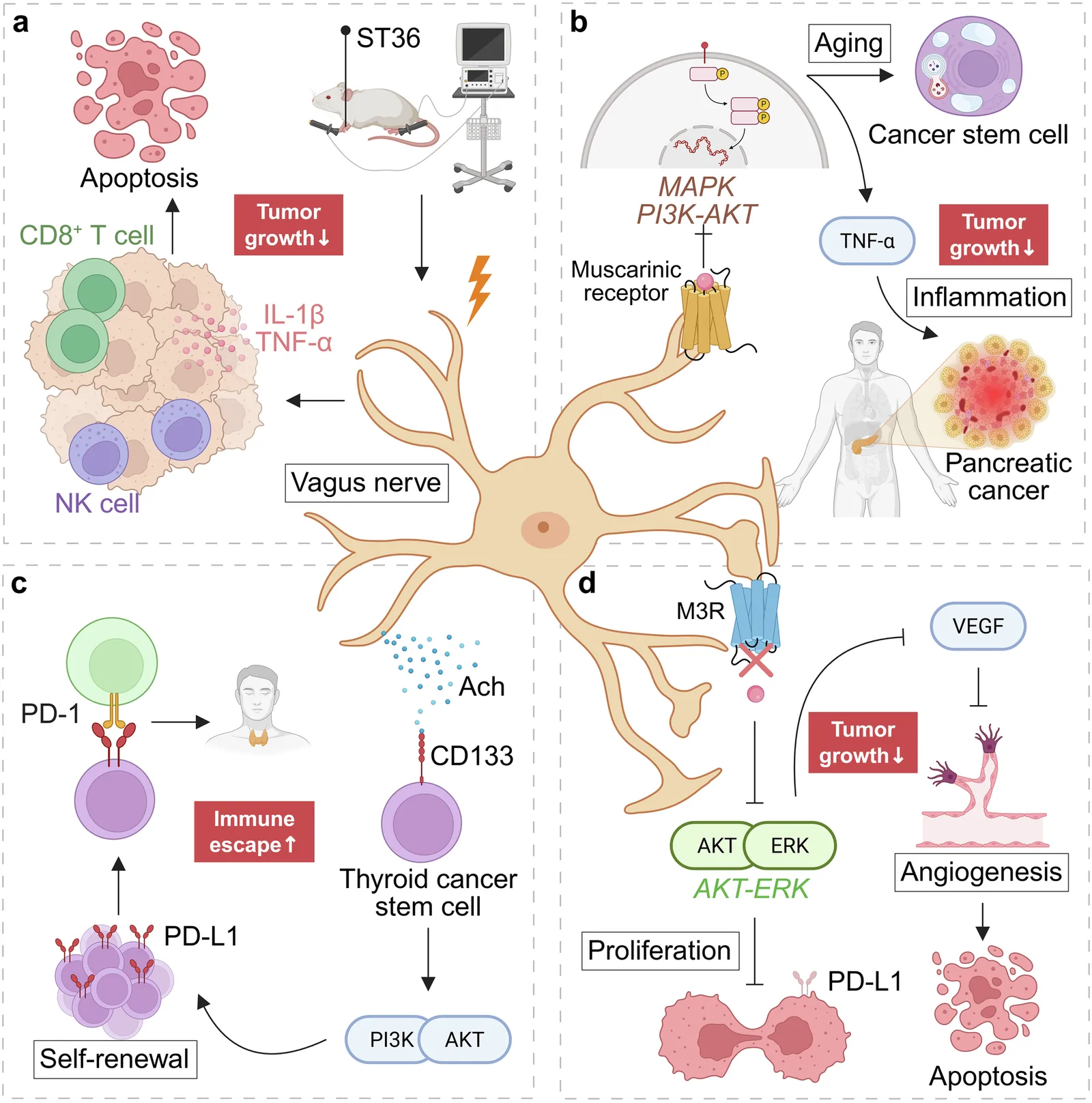
Cholinergic signaling via the vagus nerve shapes tumor progression and immune responses across cancer types. **a** Electroacupuncture stimulation at ST36 activates vagus nerve signaling, enhancing CD8⁺ T cell and natural killer (NK) cell cytotoxicity while reducing IL-1β and tumor necrosis factor-alpha (TNFα) production in the tumor microenvironment, thereby promoting tumor apoptosis and growth inhibition. **b** In pancreatic cancer, muscarinic receptor activation counteracts aging-driven and inflammation-driven tumorigenesis. Cholinergic signaling through MAPK and PI3K–AKT pathways reduces TNFα-mediated inflammation, restraining cancer stem cell expansion and tumor progression. **c** In thyroid cancer stem cells, acetylcholine produced in an autocrine manner enhances CD133⁺ cell self-renewal via PI3K–AKT signaling. This promotes PD-L1 expression, facilitating PD-1-mediated T cell suppression and immune escape. **d** Muscarinic acetylcholine receptor M3R signaling activates the AKT–ERK cascade, inhibiting vascular endothelial growth factor (VEGF)-driven angiogenesis, reducing proliferation, and downregulating PD-L1 expression. These effects collectively promote apoptosis and tumor growth suppression.

Parasympathetic signaling via the vagus nerve modulates tumor immunity, balancing inflammation and shaping antitumor responses. Subdiaphragmatic vagotomy accelerates pancreatic tumor progression by disrupting vagal anti-inflammatory signaling and promoting tumor necrosis factor-alpha (TNFα)-driven tumorigenesis. Loss of vagal input increases macrophage infiltration and intratumoral TNFα, enhancing cancer cell proliferation and migration. In TNFα-deficient mice, tumor growth is attenuated, identifying TNFα as the principal effector of vagal modulation^105^. Vagal signaling via electroacupuncture at ST36 suppresses tumor growth by reducing IL-1β and TNFα, alleviating MDSC-mediated immunosuppression, and enhancing CD8⁺ T cell and NK cell activity. These effects are abolished by vagotomy, confirming the importance of vagal activation for electroacupuncture-induced immunomodulation^106^ (Fig. 5a).

Cholinergic signaling also influences tumor biology within the TME. Muscarinic receptor-mediated cholinergic input suppresses pancreatic tumorigenesis by inhibiting MAPK and PI3K-AKT pathways, reducing cancer stem cell populations, and limiting myeloid infiltration and TNFα-driven inflammation. Loss of vagal tone or genetic deletion of CHRM1 accelerates tumor progression, whereas muscarinic receptor activation reverses these effects^107^ (Fig. 5b). In thyroid cancer, acetylcholine enhances CD133⁺ cancer stem cell self-renewal and immune evasion by activating PI3K–AKT and inducing PD-L1 expression, conferring resistance to CD8⁺ T cell-mediated cytotoxicity^108^ (Fig. 5c). In CRC, muscarinic receptor-3 (M3R) signaling supports tumor growth by reinforcing immunosuppressive crosstalk and angiogenesis, with M3R inhibition reducing tumor proliferation and immune checkpoint expression^109^ (Fig. 5d).

These findings highlight the complexity of parasympathetic regulation in cancer, with effects depending on receptor subtype and context. The vagus nerve comprises 70% sensory afferent fibers, meaning that vagotomy or electrical stimulation affects both sensory input and parasympathetic output, complicating interpretation^110^. Muscarinic acetylcholine receptors are expressed on neurons, tumor cells, macrophages, and immune cells, with subtype distribution varying across tumor types. CHRM1 activation has antitumor effects, whereas CHRM3 promotes proliferation and immune evasion^107,111^. The effects of cholinergic signaling depend on receptor subtype, downstream signaling, and model design. Using orthotopic or genetically engineered models that preserve anatomical innervation is crucial to understanding parasympathetic contributions. These considerations suggest that parasympathetic regulation in cancer is context-dependent, requiring further research with more accurate models and receptor-specific knockdown to clarify its role in tumor immunity and progression.

### Central neural circuits orchestrating tumor progression and antitumor immunity

Compared with peripheral nerves that act within or near the TME, central neural circuits regulate tumor immunity at a broader systemic level^11^. Peripheral sensory, sympathetic, and parasympathetic fibers can directly communicate with tumor, stromal, and immune cells through local release of neurotransmitters, neuropeptides, and trophic factors, thereby shaping immune cell infiltration, activation, and exhaustion within the TME^112^. By contrast, central circuits usually influence tumor immunity indirectly by modulating autonomic outflow and neuroendocrine responses, including sympathetic activity, vagal tone, and hypothalamic–pituitary–adrenal (HPA) axis activation^113,114^. Thus, peripheral nerves provide local effector signals at the tumor site, whereas central circuits integrate psychological, metabolic, circadian, and inflammatory cues and translate them into systemic immune reprogramming. Understanding this central–peripheral hierarchy is important for distinguishing local neuroimmune mechanisms from brain-driven systemic regulation of antitumor immunity.

The central nervous system (CNS) regulates tumor progression by influencing neuroimmune circuits. Activation of ventrolateral medulla catecholaminergic neurons enhances tumor growth, whereas their ablation or inhibition suppresses tumor progression through CD8⁺ T cell-dependent mechanisms^113^. In prostate cancer, neural progenitors from the subventricular zone exit the brain and infiltrate tumors, where they differentiate into adrenergic neurons that promote tumor growth and metastasis^115^. In CRC, hypothalamic oxytocin neurons suppress tumor progression by modulating peripheral sympathetic activity through the celiac-superior mesenteric ganglion. Central stimulation with celastrol enhances oxytocinergic signaling and inhibits tumor growth, an effect reversed by β2-adrenergic activation^116^. In lung cancer, retrograde neural tracing shows that tumor progression activates brainstem nuclei in the rostral ventromedial and lateral medulla, regions linked to vagal regulation, suggesting a central mechanism by which the brain modulates peripheral tumor dynamics^117^. In triple-negative breast cancer, depression-induced activation of the HPA axis increases glucocorticoids, disrupting immune homeostasis and impairing PD-L1 blockade efficacy. Inhibition of HPA signaling restores immune function and enhances immunotherapy^114^.

Beyond stress-responsive neural circuits, dopaminergic signaling adds another layer of neuroimmune regulation in cancer. Dopamine, traditionally produced by central neurons, is also synthesized within the TME by tumor and immune cells^118,119^. Studies indicate that dopamine profoundly influences immune cell differentiation and effector function, suggesting its potential to reprogram antitumor immunity^120–122^. Dopamine primarily promotes immune-mediated tumor control through dopamine receptor D5 (DRD5) and dopamine receptor D3 (DRD3) pathways^123,124^. DRD5 activation drives CD8⁺ T cell differentiation into CD103⁺ tissue-resident memory cells, enhancing immune surveillance. Loss of DRD5 impairs tissue-resident memory formation and accelerates tumor progression, whereas dopamine treatment increases CD8⁺ T cell accumulation and suppresses colorectal tumor growth. Ex vivo dopamine priming also enhances chimeric antigen receptor (CAR)-T cell efficacy, and in patients with CRC, dopamine levels correlate with CD8⁺ T cell infiltration and improved survival, highlighting a clinically relevant neural–immune axis^125^. Similarly, dopamine signaling through DRD3 enhances CD8⁺ T cell-mediated antitumor responses by promoting IL-2 production, CD25 upregulation, and T cell expansion. DRD3 deficiency weakens immune responses and impairs melanoma control^126^. Additionally, dopamine reduces immunosuppressive myeloid activity in monocytic MDSCs through D1-like receptor signaling, which suppresses nitric oxide synthesis and inactivates p-ERK and p-JNK signaling, reinforcing adaptive immunity and limiting tumor progression^94^ (Table 2).

**Table 2:** Sympathetic, parasympathetic, and central neural circuits in tumor immunoregulation.

Neuron type	Neurotransmitter or neuropeptide	Tumor type	Target cell	Immune modulation mechanism	Therapeutic potential	Ref.
Sympathetic neurons	NE (β2-ARs)	SCLC	Human SCLC cells	Promoting SCLC cell proliferation via β2-AR–PKA signaling	Performing chemical denervation or inhibiting β2-ARs slows SCLC growth and prolongs survival	^59^
		Breast cancer	Breast cancer cells	Sustaining glycolysis via HK2 stabilization	Suppressing HK2 expression by propranolol	^62^
	NE	Breast cancer	Macrophages	Inducing lymphangiogenesis via VEGFC and COX-2-dependent signaling	Blocking SNS prevents lymphatic remodeling, reduces lymphatic metastasis	^63^
	NE (β2-ARs)	CRC	CAFs, tumor cells	Driving feedforward NGF loop, activating YAP and PI3K-AKT pathways	Inhibiting Trk signaling suppresses CRC progression	^64^
	NE	Metastatic melanoma	–	Creating immunosuppressive TME, limiting ICI efficacy	Combining propranolol with PD-1 blockade enhances antitumor immunity	^67^
		–	Naive CD8⁺ T cells	Suppressing CD8^+^ T cell priming in tumor-draining lymph nodes	β-Blockers improve cancer vaccine efficacy	^72^
		Lymphoma	CD8⁺ T cells	Suppressing CD8^+^ T cell proliferation, IFN-γ production, cytotoxicity	–	^73^
	Catecholamine (β1-ARs)	Melanoma, PDAC	CD8⁺ T cells	Promoting CD8^+^ T cell exhaustion, suppressing cytokine production	Blocking β1-ARs reduces CD8^+^ T cell exhaustion, synergizes with ICI	^65^
	NE (β2-ARs)	Multiple cancers	T cells	Impairing TCR signaling and metabolism, inducing exhaustion in T cells	Improving effector phenotype of TILs by propranolol	^74^
		–	CD8⁺ T cells	Suppressing CD8⁺ T cell effector function	Reducing β-AR signaling improves anti-PD-1 therapy	^69^
		–		Disrupting glucose uptake and metabolic reprogramming	–	^68^
	NE (β1-ARs)	Hepatocellular carcinoma	MAIT_reg_ cells	Expanding regulatory MAIT cells, suppressing immunity	–	^75^
	Epinephrine (β2-ARs)	Breast cancer	Macrophages	Promoting M2 polarization, reinforcing immunosuppressive niche	–	^76^
	Catecholamines	Breast cancer	NK cells	Suppressing NK cytotoxicity, facilitating metastasis	Sympathetic blockade or adrenal demedullation restores NK activity, reduces MADB106 metastasis	^77^
	NE (α-AR)	–	Myeloid cells	Maintaining myeloid maturation, preventing suppressive MDSCs	Blocking α-AR impairs immunity and induces T_reg_ cells	^111^
	NE (β2-ARs)	–	MDSCs	Reprogramming metabolism to OXPHOS and FAO, increasing PGE2-mediated immunosuppression	–	^112^
		–	MDSCs	Enhancing MDSCs survival via STAT3, arginase-I, PD-L1, and apoptosis resistance	β2-ARs blockade reduces immune evasion and enhances immunotherapy	^78^
	NE	PDAC	CD163⁺ macrophages	Inducing CD163⁺ macrophage infiltration	Sympathetic nerve ablation accelerates tumor growth and dissemination	^79^
	NE (α2-AR)	CRC	Tumor cells, CD8⁺ T cells	Promoting chemokine induction and CD8⁺ T cell recruitment	Facilitating CRC malignant progression by neurodegeneration	^80^
		Multiple tumor models	T cells, macrophages	Enhancing antigen presentation and T cell infiltration	Using α2-AR agonists to boost ICI efficacy	^81^
Vagus nerve	–	PDAC	TAMs	Suppressing TNFα-driven tumorigenesis, reducing macrophage infiltration	Activating vagal signaling to inhibit inflammation and tumor growth	^82^
	–	Breast cancer	CD8⁺ T cells, NK cells, MDSCs	Attenuating inflammation, boosting cytotoxic immunity, and suppressing MDSCs	Stimulating vagus nerve to enhance antitumor immunity	^83^
	ACh (CHRM1)	PDAC	Pancreatic cancer cells, myeloid cells	Suppressing MAPK and PI3K–AKT pathways, depleting cancer stem cells (CSCs), limiting inflammation	Activating muscarinic signaling to suppress tumorigenesis	^84^
	ACh	Thyroid cancer	Thyroid CSCs, CD8⁺ T cells	Promoting CSC stemness and PD-L1 expression by activating CD133–Akt axis	Blocking AChR to limit CSC-driven immune escape	^85^
Cholinergic neurons	ACh (M3R)	CRC	CT-26 cells, CD4⁺ and CD8⁺ T cells	Enhancing CT-26 cell proliferation, PD-L1 and PD-L2 expression, angiogenesis	Blocking M3R to suppress tumor growth and immunosuppression	^86^
VLM neurons	Catecholamines	–	CD4⁺ and CD8⁺ T cells	Suppressing the infiltration and accumulation of CD4^+^ and CD8^+^ T cells	Ablating VLM neurons to slow tumor growth	^89^
CeM^CRH^ neurons	CRH	Breast cancer	–	Driving sympathetic activity, NE release, suppressing antitumor immunity	Targeting CRH circuit or anxiolytics to disrupt stress-induced progression	^113^
DAergic neurons	DA (DRD5)	CRC	CD8⁺ T cells	Promoting TRM differentiation and effector function	Improving CAR-T cell efficacy by dopamine priming	^101^
	DA (DRD3)	Melanoma	CD8⁺ T cells	Enhancing CD8⁺ T cell IL-2 production, CD25 upregulation, and effector expansion	Activating DRD3 to reinforce T cell-mediated immunity	^102^
	DA (D1-like receptor)	–	M-MDSCs	Reducing immunosuppression by inhibiting NO synthesis	Enhancing antitumor immunity through DA and D1 receptor agonists	^71^

*AR* adrenergic receptor, *CAF* cancer-associated fibroblast, *CAR-T* chimeric antigen receptor-T, *CRC* colorectal cancer, *DA* dopamine, *DRD3* dopamine receptor D3, *DRD5* dopamine receptor D5, *FAO* fatty acid oxidation, *HK2* hexokinase-2, *ICI* immune checkpoint inhibitor, *IFN* interferon, *MAIT* mucosa-associated invariant T, *MDSC* myeloid-derived suppressor cell, *M3R* muscarinic receptor-3, *NGF* nerve growth factor, *NK* natural killer, *NO* nitric oxide, *OXPHOS* oxidative phosphorylation, *PDAC* pancreatic ductal adenocarcinoma, *PKA* protein kinase A, *SCLC* small-cell lung cancer, *SNS* sympathetic nervous system, *TAM* tumor-associated macrophage, *TCR* T cell receptor, *TIL* tumor-infiltrating lymphocyte, *TME* tumor microenvironment, *TNFa* tumor necrosis factor-alpha, *Treg* regulatory T, *TRM* tissue-resident memory, *VLM* ventrolateral medulla, *CeM* central medial amygdala, *CRH* corticotropin-releasing hormone.

### Neuro–tumor communication in brain malignancies

Unlike peripheral solid tumors, neoplasms arising within the CNS exist in a highly specialized neuroglial milieu that fosters extensive communication among tumor cells, neurons, and glia. The electrical and chemical activity of the brain provides unique avenues through which tumor cells interact with neural circuits and reshape local physiology. These interactions encompass neurotransmitter-driven signaling, neural activity-dependent reprogramming, and neuroimmune modulation^127^.

Retrograde viral tracing shows extensive neuron–tumor communication, with cholinergic neurons promoting glioblastoma progression. Radiotherapy increases neuronal activity and enhances this connectivity, whereas neuronal inhibition boosts treatment efficacy. Genetic ablation of tumor-connected neurons halts tumor growth, highlighting neuron-derived activity as crucial for glioblastoma invasiveness and therapy response^128^. Glioblastoma integrates into local and long-range neural circuits, with glutamatergic and cholinergic signaling driving calcium oscillations and transcriptional reprogramming through CHRM3, promoting tumor cell motility. Inhibition of this acetylcholine pathway reduces invasion and prolongs survival^129^.

Neuronal activity in glioma cells enhances BDNF–TrkB–CaMKII signaling, promoting tumor proliferation through AMPA receptor trafficking and membrane depolarization. Disrupting BDNF release or TrkB function suppresses tumor progression and extends survival^130^. High-grade gliomas remodel neural circuits, creating abnormal connectivity between tumor-infiltrated and cognitive brain regions. Neuronal activity within these networks drives tumor growth, whereas glioma cells secrete thrombospondin-1 to enhance neuron–tumor interactions. Inhibiting thrombospondin-1 with gabapentin reduces proliferation, and tumor–brain connectivity correlates with cognitive impairment and poor survival^131^. Neuronal expression of calmodulin-dependent kinase kinase 2 (CaMKK2) sustains an immunosuppressive TME in glioblastoma by promoting resistance to ICIs. CaMKK2 activity induces CD8⁺ T cell exhaustion and limits CD4⁺ T cell expansion, linking neuronal signaling to immunotherapy resistance^132^. In H3K27M-altered diffuse midline gliomas, GABAergic neurons form tumor-promoting connections through depolarizing GABA_A_ receptor signaling, enhancing tumor growth^133^. Brain tumor cells also exploit neuron–microglia signaling to support growth by mimicking activity-dependent cues that recruit microglia. Elevated intracellular Ca^2+^ in AKT1^+^ cells triggers ATP release, engaging P2RY12 on microglia, sustaining interactions. Disrupting calcium signaling, ATP release, or P2RY12 halts microglial recruitment and suppresses tumor growth^134^.

### Boundary conditions shaping context-dependent neuroimmune regulation

The outcome of neuroimmune signaling is highly context-dependent; however, this context can be more precisely defined by a set of key boundary conditions that shape both signal delivery and cellular responsiveness. First, neural architecture, including innervation density, neuronal subtype composition, and the spatial localization of nerve fibers within the tumor core, invasive margin, perineural niche, or tertiary lymphoid structures, determines which immune populations are exposed to neural cues and the extent of that exposure^67,135^. Second, anatomical site and tumor type establish the baseline neural and immune landscape, thereby influencing whether sensory, sympathetic, parasympathetic, or central autonomic inputs predominate^136^. Third, receptor cell pairing represents a critical determinant of functional outcome: the same neurotransmitter or neuropeptide may elicit divergent, or even opposing, effects depending on whether its receptors are expressed by tumor cells, CD8^+^ T cells, macrophages, MDSCs, SCs, or stromal compartments^14^. Fourth, the pre-existing immune state of the TME, such as T cell-inflamed versus immune-cold phenotypes, exhausted T cell enrichment, NK cell infiltration, or myeloid dominance, further conditions whether neural inputs amplify immune surveillance or reinforce immunosuppressive circuits^137,138^. Finally, signal dynamics — including acute versus chronic activation, local versus systemic exposure, stress intensity, circadian timing, and treatment-induced remodeling — govern the magnitude, duration, and directionality of neuroimmune effects^11^.

Importantly, these variables do not operate in isolation but act in combination to determine the net functional outcome of neuroimmune signaling within a given tumor context. This integrative framework helps reconcile apparently contradictory observations across studies and shifts the interpretation of neuroimmune pathways from binary (pro-tumor versus antitumor) classifications to context-contingent regulatory states. From a translational perspective, explicitly defining these boundary conditions may enable more predictive and context-guided selection of neuroimmune targets, linking therapeutic intervention to the underlying signaling landscape. Such an approach is consistent with emerging principles in cancer immunotherapy, in which target prioritization increasingly depends on signaling context rather than pathway identity alone, thereby improving the precision and clinical relevance of neuroimmune modulation strategies^139^.

## Glial control of immune evasion and TME plasticity

Although neurons are known regulators of neuroimmune interactions in cancer, emerging evidence shows that glial cells are equally crucial in shaping tumor–immune dynamics. Glial cells not only maintain neural integrity but also actively remodel the TME by releasing neurotrophic and immunomodulatory factors that create immunosuppressive niches, promoting tumor progression and therapeutic resistance^140^.

**Fig. 6:**
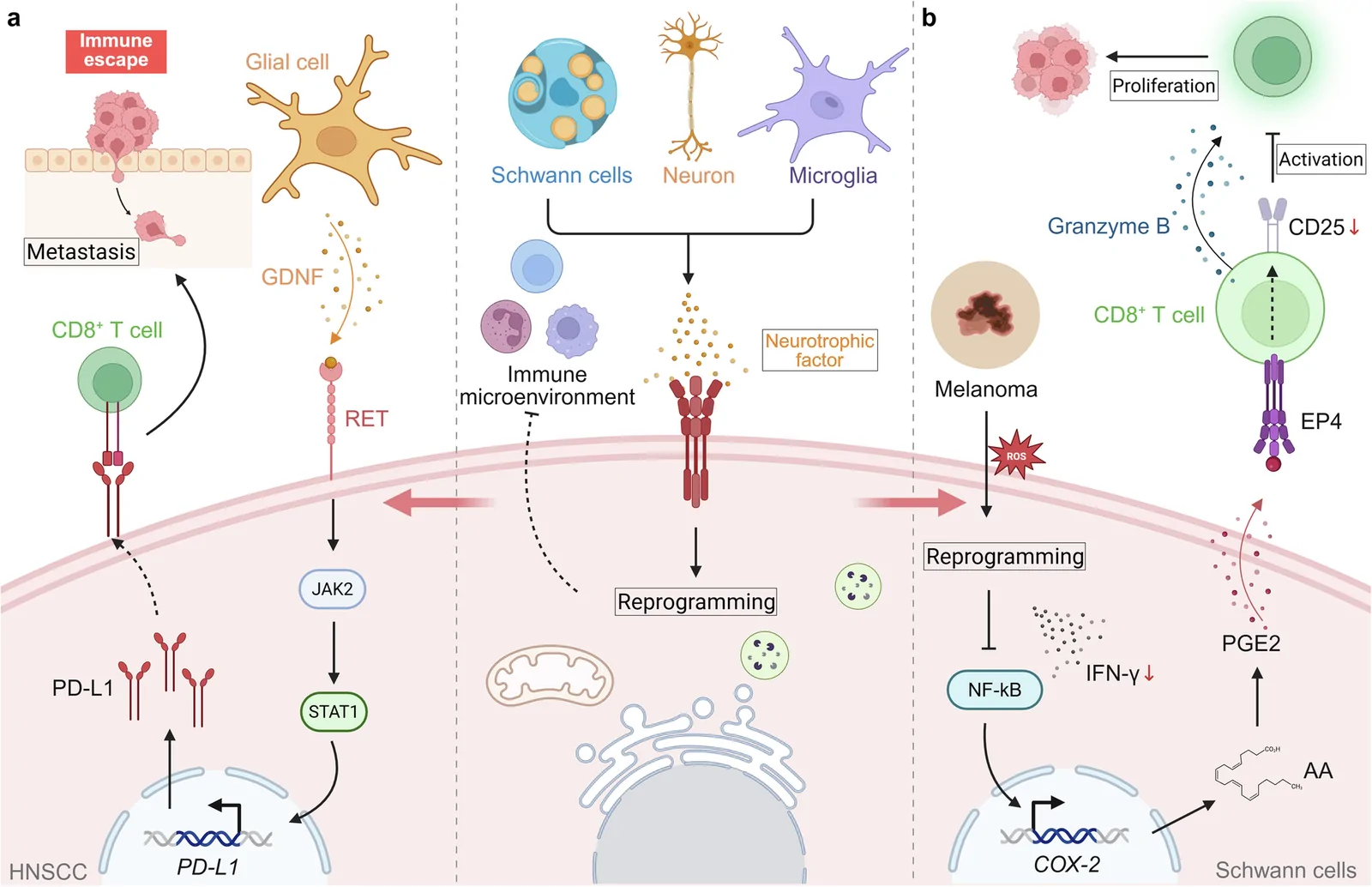
Glial cells reshape the tumor–immune microenvironment to drive immune escape. Neurons, Schwann cells, and microglia secrete neurotrophic factors that reshape the immune microenvironment and drive tumor cell reprogramming. **a** In head and neck squamous cell carcinoma (HNSCC), glial cell-derived neurotrophic factor (GDNF) activates RET signaling in tumor cells, leading to JAK2-STAT1-dependent induction of PD-L1. Elevated PD-L1 suppresses CD8⁺ T cell cytotoxicity, facilitating immune evasion and metastatic dissemination. **b** In melanoma, Schwann cell-derived arachidonic acid is metabolized to PGE2, which signals via EP4 on CD8⁺ T cells. This reduces CD25 expression, impairs activation and granzyme B production, and limits cytotoxicity. Concurrently, reactive oxygen species (ROS) activates nuclear factor (NF)-κB in tumor cells, inducing COX-2 and suppressing interferon-γ (IFNγ) expression, thereby reinforcing immune escape.

In HNSCC, glial cell line-derived neurotrophic factor (GDNF) drives PD-L1 expression via JAK2–STAT1 signaling, correlating with increased perineural invasion and poor patient outcomes. GDNF enhances neural invasion and confers resistance to NK cell-mediated cytotoxicity, with JAK2 inhibition reversing PD-L1 induction, thus linking glial activity to immune evasion in the perineural niche^141^ (Fig. 6a). Similarly, during pancreatic tumorigenesis, SCs undergo reprogramming that stimulates sympathetic nerve sprouting and neural remodeling. SCs upregulate GDNF, whereas sympathetic neurons increase GDNF receptor expression and neurite extension, processes reversed by SC-specific *Gdnf* deletion^142^.

Beyond neurotrophic signaling, SCs influence the TME by initiating immunosuppressive programs. In melanoma, peritumoral SCs adopt an anti-inflammatory phenotype, producing lipid mediators such as PGE2 and lipoxins, which inhibit T cell activation via EP4 signaling, dampening both local and systemic antitumor immunity^143^ (Fig. 6b). In lung cancer, SCs promote macrophage M2 polarization through secretion of chemokines such as CCL2, CXCL5, and CXCL8, which in turn enhance tumor proliferation, creating a neuro–immune–tumor loop that fosters malignant progression^144^. In PDAC, SCs infiltrate the TME, reprogramming tumor and stromal cells toward aggressive phenotypes. Through midkine and IL-1α signaling, they induce tumor cells to transition to a basal-like state and alter CAFs to an inflammatory subtype^145^. Tissue-resident SCs localize to the perivascular niche, promoting angiogenesis and immune escape, with their genetic ablation reducing tumor growth, enhancing lymphocyte infiltration, and diminishing immunosuppressive populations^146^.

## Neuron-independent neural signaling in tumor–immune evasion

Neuron-independent neural signaling involves non-neuronal cells, such as immune and tumor cells, activating neurotransmitter receptors (for example, adrenergic, cholinergic, and peptidergic receptors). Unlike traditional nerve fiber-mediated regulation, in which neural fibers directly transmit signals via neurotransmitter release and action potentials, this mechanism enables tumor and immune cells to influence TME through neurotransmitter-based communication, even without direct neural innervation. This emerging form of signaling has gained increasing attention for its role in immune evasion and tumor progression, opening new avenues for therapeutic intervention beyond conventional neural pathways.

### Non-neuronal neurotransmitter–receptor circuits in the TME

**Fig. 7:**
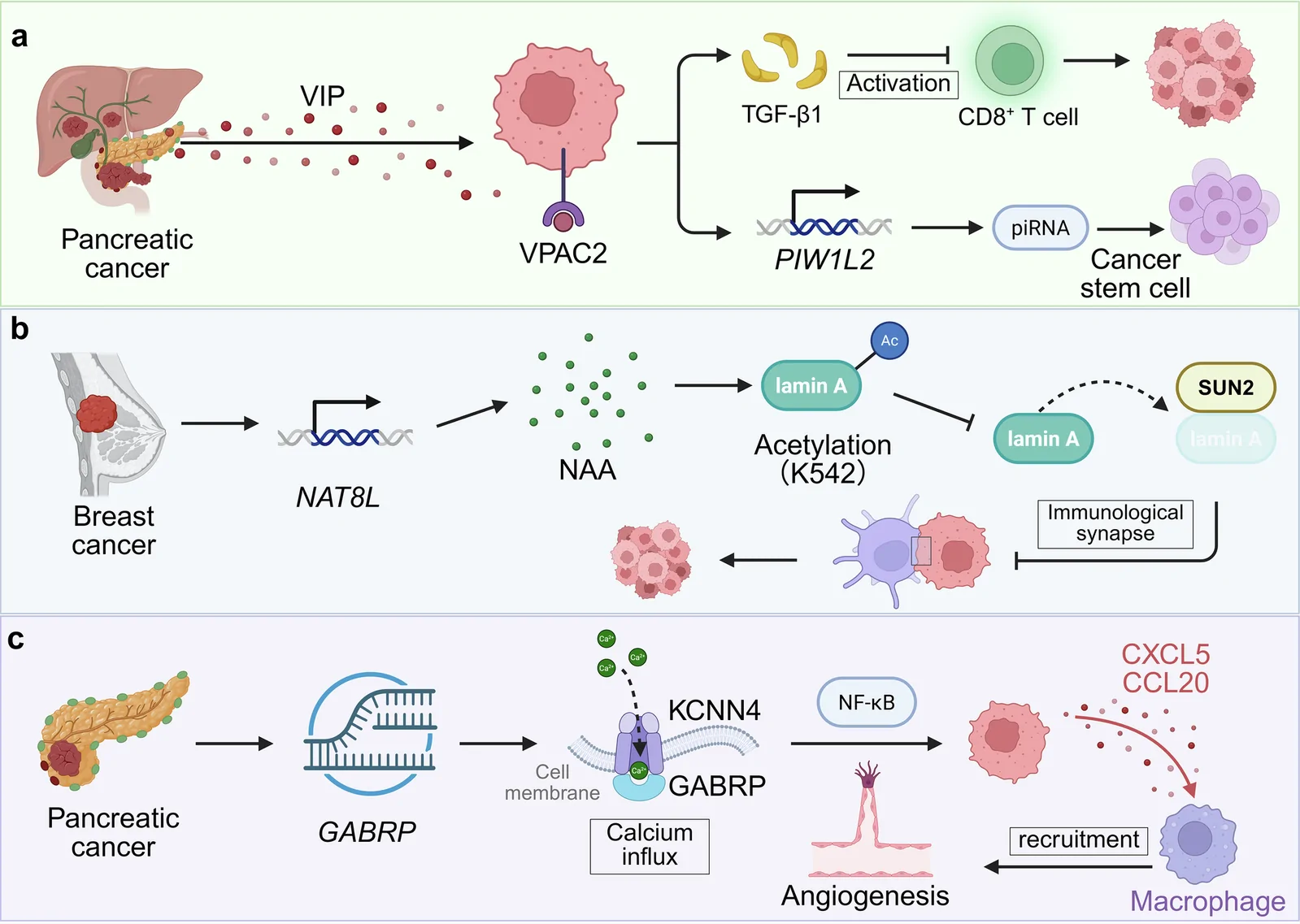
Tumor cells mimic neuronal signaling pathways to reshape immunity and support cancer progression. **a** In pancreatic cancer, tumor cells exploit VIP–VPAC2 signaling to activate transforming growth factor-beta 1 (TGF-β1) and induce PIWIL2 expression, driving piRNA-mediated cancer stemness. TGF-β1 simultaneously dampens CD8⁺ T cell activation, weakening antitumor immunity. **b** In breast cancer, NAT8L-driven accumulation of *N*-acetylaspartate (NAA) promotes lamin A acetylation at K542. This modification disrupts SUN2–lamin A interactions and impairs immunological synapse formation, ultimately attenuating T cell-mediated immune surveillance. **c** In pancreatic cancer, tumor cells mimic GABAergic signaling via GABRP, which cooperates with KCNN4 to enhance calcium influx and nuclear factor (NF)-κB activation. This axis promotes angiogenesis and induces CXCL5 and CCL20 secretion, recruiting macrophages that reinforce an immunosuppressive microenvironment.

Within the TME, neurotransmitters are not only derived from neural inputs but also synthesized and released by non-neuronal cells, including platelets, B cells, tumor-infiltrating immune populations, and even tumor cells. Tumors hijack these neurotransmitter–receptor circuits to suppress cytotoxic lymphocyte activity and maintain an immunosuppressive environment, extending the neuroimmune axis beyond the nervous system^147^. For instance, elevated VIP levels in PDAC and patient plasma suppress antitumor immunity by activating VIP receptors on T cells. Inhibiting VIP-receptor signaling enhances T cell infiltration, reverses exhaustion, and synergizes with PD-1 blockade to induce tumor regression and durable immune memory^148^. Conversely, autocrine VIP–VIP receptor 2 (VPAC2) signaling in pancreatic cancer cells promotes clonogenic growth and immune evasion through TGFβ1-mediated T cell suppression. High VIP–VPAC2 co-expression correlates with poor survival, whereas VPAC2 loss increases sensitivity to anti-PD-1 therapy^149^ (Fig. 7a). Non-neuronal metabolites also influence tumor immunity. Activated B cells release GABA, driving monocyte differentiation into IL-10-producing macrophages that suppress CD8⁺ T cell cytotoxicity. Loss of B cells or inhibition of GABA synthesis enhances antitumor immunity in vivo, revealing a neuroimmune metabolic circuit in which B cells use a classical neurotransmitter to dampen immune activation^150^. Similarly, 5-HT, largely produced by platelets, promotes immune evasion by enhancing PD-L1 expression through serotonylation and restricting CD8⁺ T cell infiltration. Serotonin-deficient mice show reduced tumor growth and increased CD8⁺ T cell accumulation, with lower PD-L1 expression in both pancreatic and CRC models. Pharmacological depletion of serotonin using fluoxetine or TPH1 inhibitors synergizes with PD-1 blockade to enhance tumor control^151^.

### Neuron-independent neural mimicry and pseudo-neural programs

Beyond utilizing host-derived neurotransmitters, the TME can create pseudo-neural pathways that mimic neuronal communication. This neuron-independent neural mimicry allows tumor and stromal cells to replicate features of the nervous system, distorting immune communication, disrupting effector coordination, and supporting tumor survival. This mechanism of immune evasion highlights an overlooked aspect of tumor plasticity in reshaping neuroimmune networks^152,153^.

Tumor cells mimic a neural-like mechanism by upregulating NAT8L and its metabolite *N*-acetylaspartate (NAA), which impair cytotoxic lymphocyte function. NAA disrupts immunological synapse formation by acetylating lamin A, blocking its interaction with SUN2, and preventing lytic granule polarization. Elevated NAT8L expression correlates with resistance to anti-HER2 and anti-PD-L1 therapies, revealing a neuroimmune mimicry mechanism for immune evasion and positioning NAT8L as a potential therapeutic target^152^ (Fig. 7b). In PDAC, the GABA receptor π subunit (GABRP) promotes tumor progression through a neurotransmitter-independent mechanism. GABRP interacts with KCNN4 to enhance Ca^2+^ influx, activate NF-κB signaling, and upregulate CXCL5 and CCL20 to recruit macrophages. Depletion of macrophages abolishes GABRP’s tumor-promoting effects, confirming its role in immune remodeling within the TME^154^ (Fig. 7c and Table 3).

**Table 3:** Mechanisms of neuron-independent neural signaling in tumor–immune evasion.

Mechanism type	Key molecule	Source of signal	Impact on immune	Disease type	Potential target	Ref.
Increasing TGFβ1 expression Upregulating Piwil2 expression	VIP	Pancreatic cancer cells	T cell function↓	PDAC	VPAC2	^132^
Inducing anti-inflammatory IL-10^+^ macrophages	GABA	B cells	Reducing IFN-γ, TNFα, and granzyme B	Colon adenocarcinoma	GAD67	^133^
Upregulating PD-L1 expression	5-HT	Platelet	CD8^+^ T cell function↓	CRC	TPH1 SERT	^134^
Disrupting immunological synapse formation	NAA	Breast cancer cells	Perforin, granzyme B, and IFN-γ↓	Breast cancer	NAT8L	^135^
Inhibiting NK cell cytotoxicity via the CSK-ZAP70-NF-κB axis	L-lactate	–	IFN-γ and granzyme B transcription↓	Melanoma	GPR132	^138^
Calcium influx activates NF-κB pathway	Ca^2+^	Pancreatic cancer cells	CD8⁺ T cell infiltration↓	PDAC	GABRP	^137^

*CRC* colorectal cancer, *GABRP* GABA receptor p subunit, *IFN* interferon, *NAA* N-acetylaspartate, *NK* natural killer, *PDAC* pancreatic ductal adenocarcinoma, *TGFß1* transforming growth factor-beta 1, *TNFa* tumor necrosis factor-alpha, *5-HT 5*-hydroxytryptamine.

### Receptor-dependent, ligand-dependent, and time-dependent control of neuroimmune signaling

Neural regulation of tumor immunity operates across multiple levels, involving peripheral and central neural inputs, glial remodeling, tumor-induced neural rewiring, and neuron-independent neuromodulatory signaling. Across these settings, similar neural cues can produce markedly different immune outcomes, indicating that neuroimmune signaling is shaped by how a given signal is received, amplified, and sustained within the tumor microenvironment. This variability is largely determined by the receptor-bearing cells that encounter the signal, the local availability of the ligand, and the duration or recurrence of signaling.

Receptor subtype determines how the same class of neural signal is translated by immune or tumor-associated cells. Adrenergic signaling provides the clearest example. β1-AR signaling links sympathetic catecholamine exposure to CD8^+^ T cell exhaustion^87^. β2-AR signaling suppresses T cell receptor-mediated CD8^+^ T cell effector function in both human and mouse T cells^92^. By contrast, α2-AR activation can trigger immune-mediated tumor rejection in immunocompetent tumor models^104^. A similar distinction appears in cholinergic signaling, in which muscarinic receptor activation suppresses pancreatic tumorigenesis and cancer stemness^107^, whereas M3R blockade in CRC reduces tumor growth together with immunosuppressive, cholinergic, and angiogenic markers^109^. Beyond autonomic neurotransmitters, CGRP–RAMP1 signaling further illustrates the importance of receptor-expressing immune cell type. In melanoma, nociceptor-derived CGRP acts on RAMP1-expressing CD8^+^ T cells and promotes T cell exhaustion^71^, whereas RAMP1^+^ B cells suppress CD8^+^ T cell cytotoxicity and contribute to resistance to neoadjuvant anti-PD-1-based therapy in esophageal squamous cell carcinoma^79^. These examples indicate that neuroimmune output is determined by the specific ligand–receptor–cell pairing, rather than by neurotransmitter or neuropeptide identity alone.

Ligand availability then determines how strongly these receptor-defined pathways are engaged. Norepinephrine suppresses CD8^+^ T cell cytokine production through β2-AR signaling, with the magnitude of suppression related to ligand exposure^92^. β-Adrenergic stimulation also limits activation-associated metabolic reprogramming in CD8^+^ T cells, linking adrenergic signal intensity to impaired glycolytic and mitochondrial adaptation^91^. Serotonin follows the same quantitative logic in tumor immunity. Peripheral serotonin attenuation increases CD8^+^ T cell accumulation and improves immune checkpoint blockade in murine tumor models^151^. In NSCLC, neural infiltration increases intratumoral 5-HT, which promotes glycolysis and an immunosuppressive microenvironment through PI3K-Akt-mTOR signaling^75^. Recent work on the intratumoral serotonin axis further shows that altered serotonin handling can restrain CD8^+^ T cell-mediated antitumor immunity^155^. VIP-mediated signaling also illustrates this principle, because VIP receptor blockade enhances T cell activation and improves response to PD-1 blockade in PDAC models^156^. Thus, neural ligands function less as binary switches than as exposure-dependent signals that can push an existing receptor–cell circuit toward activation or suppression.

Temporal dynamics further determine which phase of the antitumor–immune response is exposed to neural regulation. During immune priming, β-adrenergic blockade enhances CD8^+^ T cell priming and improves cancer vaccine efficacy^95^. Under chronic adrenergic stress, the same broad sympathetic axis instead promotes metabolic dysfunction and an exhausted phenotype in tumor-infiltrating T cells^97^. Temporal control is also relevant in sensory circuits, as repeated activation of TRPV1^+^ sensory neurons facilitates tumor growth and shifts the tumor-infiltrating immune landscape toward reduced T cell infiltration and increased immunosuppressive myeloid components^72^. Together, these dimensions define a unified model in which neuroimmune signaling outcomes are determined by the integrated effects of receptor subtype, ligand availability, and temporal dynamics, rather than by any single factor alone.

## Challenges in decoding neuroimmune crosstalk in cancer

**Fig. 8:**
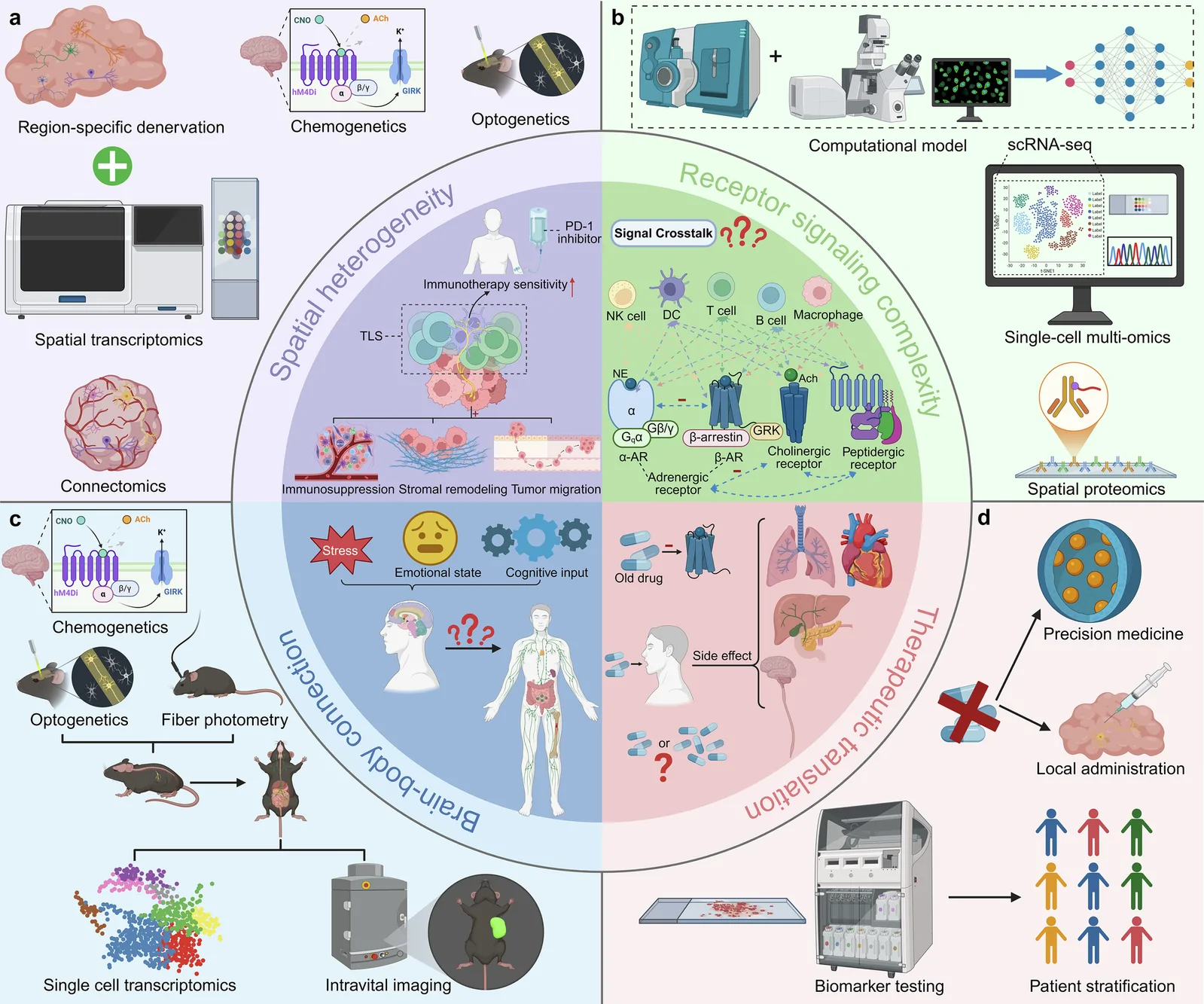
Challenges and future directions in dissecting neuroimmune interactions in cancer. **a** Deciphering how distinct neural inputs shape local immune landscapes remains a major challenge. Tools such as region-specific denervation, chemogenetics, optogenetics, spatial transcriptomics, and connectomics will enable high-resolution mapping of tumor–nerve–immune interactions. **b** Tumor and immune cells integrate signals from multiple neurotransmitter and neuropeptide receptors, including adrenergic, cholinergic, and peptidergic pathways, with extensive crosstalk. Emerging approaches such as single-cell multi-omics, spatial proteomics, and computational modeling are essential to resolve this complexity. **c** Cognitive inputs, stress, and emotional state influence peripheral tumor immunity via central neural circuits. Advanced models combining optogenetics, chemogenetics, fiber photometry, intravital imaging, and single-cell transcriptomics will be critical for linking systemic physiology to tumor outcomes. **d** Harnessing neuroimmune crosstalk for therapy requires precision strategies that minimize off-target effects. Integration of patient stratification, biomarker testing, local drug administration, and repurposing of neuromodulatory agents may enable individualized treatment while avoiding adverse systemic effects. AR, adrenergic receptor; DC, dendritic cell; NK, natural killer; scRNA-seq, single-cell RNA sequencing; TLS, tertiary lymphoid structure.

Despite progress in understanding neural influences on the TME, challenges remain, especially in spatial heterogeneity, receptor-specific signaling, and central–peripheral integration. These issues require high-resolution mapping, functional perturbation, and drug development. A key challenge is unraveling the spatial heterogeneity of neural–immune interactions within tumors. Current studies treat nerve sprouting uniformly, but the impact of neuronal inputs varies based on anatomical location and cellular context. For instance, in bladder cancer, nerve inputs in the tumor core promote NK cell dysfunction, whereas inputs in tertiary lymphoid structures enhance PD-1 expression and immunotherapy response^135^. To address this, integrating spatial transcriptomics, connectomics, and region-specific denervation or optogenetics can link spatially restricted innervation to immune outcomes, potentially identifying tumor neural architecture as a biomarker for immunotherapy response (Fig. 8a).

Adrenergic, cholinergic, and peptidergic signals engage receptor families that exert context-dependent effects on immunity. Creating a receptor–cell–function atlas is a major challenge, mapping receptor expression across immune cells and how it evolves with stress, inflammation, or treatment. This will require integrating single-cell multi-omics, spatial proteomics, and computational models to map receptor–function relationships under physiological and therapeutic conditions. This approach could enable precision neuromodulation to target immunosuppressive pathways and enhance immunostimulatory ones (Fig. 8b).

Although peripheral nerve–tumor interactions have been studied, the contribution of central neural circuits remains underexplored. The CNS influences peripheral immunity through autonomic outputs and integrates emotional and cognitive inputs that affect immune tone^157^. Preclinical models show that activation of brainstem catecholaminergic neurons accelerates tumor growth by enhancing sympathetic output and suppressing cytotoxic lymphocytes^113^. Methods such as optogenetics and chemogenetics can link brain activity with immune reprogramming^158^. Understanding these brain–tumor loops may lead to neuromodulation-based therapies and personalized immunotherapies (Fig. 8c).

Topical application of Pliaglis enhances anti-PD-1 therapy and suppresses melanoma by shifting myeloid populations and boosting inflammatory markers^159^. Neuromodulatory agents could reprogram the tumor–immune interface to overcome immunotherapy resistance. However, challenges remain in optimizing doses of anesthetics or β-blockers and developing targeted compounds to minimize toxicity^160–162^. Biomarkers such as tumor neural sprouting, plasma catecholamine levels, and neuroreceptor expression on tumor or immune cells can aid in patient selection^28,163–165^. Integrating these biomarkers into testing pipelines would enable precision neuroregulation in cancer immunotherapy (Fig. 8d).

Translating neuroimmune-targeting strategies into clinical oncology requires a more rigorous assessment of feasibility, specificity, safety, and patient selection than is currently appreciated. Among the approaches discussed, β-adrenergic blockade represents the most clinically accessible entry point, largely due to the extensive clinical experience, favorable cost profile, and well-characterized pharmacology of β-blockers. Retrospective analyses and early-phase studies, including combinations such as propranolol with ICIs, suggest a potential capacity to modulate antitumor immunity. However, these observations remain context-dependent and are not yet sufficient to establish causality or generalizability across tumor types^166,167^. The heterogeneity of AR expression, variability in tumor innervation, and differences in baseline immune status are likely to influence therapeutic outcomes, underscoring the need for stratified clinical evaluation rather than broad application^168^. In comparison, neuromodulatory strategies, including vagus nerve stimulation, electroacupuncture, and central circuit modulation, offer a mechanistically attractive means of regulating systemic and local immune responses^106,110^. Nevertheless, their translation into oncology remains limited by several unresolved issues. These include incomplete mapping of tumor-relevant neural circuits, insufficient understanding of stimulation parameters required to achieve sustained immunological effects, and uncertainty regarding the durability and reversibility of such interventions. Moreover, the bidirectional nature of neuroimmune communication raises the possibility that perturbation of one circuit may lead to compensatory activation of others, thereby attenuating therapeutic efficacy or generating unanticipated outcomes.

A major and often underappreciated challenge is the risk of systemic off-target effects. Neural signaling pathways that influence tumor immunity are deeply integrated with the regulation of cardiovascular function, metabolism, neuroendocrine homeostasis, pain perception, and behavior^11,157^. As a result, systemic modulation — particularly when sustained — may lead to unintended physiological consequences that are difficult to predict based solely on tumor-centric models. This is especially relevant in patients with comorbidities or in settings requiring long-term intervention. Therefore, strategies that enable spatially or temporally restricted modulation, such as localized delivery or context-responsive systems, may offer advantages but also introduce additional layers of complexity in design and validation. Equally important is the issue of patient stratification, as the efficacy of neuroimmune-targeting interventions is unlikely to be uniform across patient populations^137^. Potentially actionable biomarkers include the expression of neuroreceptors on tumor and immune cells, such as adrenergic, cholinergic, peptidergic, and dopaminergic receptors, which can be assessed using transcriptomic, proteomic, or spatial profiling approaches^168^. In addition, structural and microenvironmental features, including tumor innervation density, perineural invasion, and the spatial organization of nerve fibers, may provide insight into the degree of neural involvement. Circulating neuroendocrine mediators, such as catecholamines, cortisol, CGRP, VIP, and serotonin, may further reflect systemic neuroimmune states^11^. Integrating these parameters into clinical trial design could improve patient selection and enable a more precise evaluation of therapeutic benefit. Taken together, although neuroimmune modulation offers a promising extension of current immunotherapeutic paradigms, its clinical translation requires a more cautious and structured approach. Future studies should prioritize biomarker-driven trial designs, incorporate longitudinal monitoring of both neural and immune parameters, and explicitly address the balance between therapeutic efficacy and systemic physiological impact. Such efforts will be essential to determine whether neuroimmune-targeting strategies can move beyond mechanistic plausibility toward reproducible clinical benefit.

## Conclusions and perspectives

The intersection of neuroscience and oncology has shown that the nervous system is a key component of tumor–immune crosstalk. Neurons and glial cells act as dynamic regulators of immune surveillance, inflammatory tone, and stromal organization through neurotransmitters, neuropeptides, and trophic factors. Tumor cells reprogram neural and glial states, creating reciprocal neuro–tumor circuits that enhance immunosuppression, foster invasion, and promote therapeutic resistance. Immune cells also co-opt neuromodulatory receptors and G protein-coupled signaling, further diversifying tumor–immune evasion strategies.

Critical challenges remain. Neural effects on tumor immunity are context-dependent, shaped by receptor specificity, spatial localization, and TME architecture. Central–peripheral integration adds complexity, as brain circuits influence peripheral immunity through stress and emotional states. Addressing these requires integrating multi-omics, connectomic mapping, and perturbation strategies, alongside computational models to capture neuroimmune dynamics. Developing neuromodulatory therapeutics, both repurposed and next-generation agents, will be crucial, guided by biomarkers that stratify patients based on neural activity and immune response.

Elucidating the neuroimmune axis opens a new translational frontier in cancer biology. However, because neural regulation of tumor immunity is highly context-dependent, future interventions should move beyond broad neuromodulation toward precision strategies that selectively inhibit immunosuppressive neural signals or enhance immunostimulatory circuits according to tumor type, neural subtype, receptor expression, and spatial organization of the TME. Such context-guided approaches may expand the therapeutic window of ICI and support more durable antitumor immunity.

## Availability of data and materials

The data generated are included within the manuscript.
